# Autophagy‐Independent Function of ATG‐18 Is Essential for Gonadal Longevity in *Caenorhabditis elegans*

**DOI:** 10.1111/acel.70454

**Published:** 2026-03-29

**Authors:** Tatsuya Shioda, Ittetsu Takahashi, Makoto Horikawa, Taiichi Osumi, Takayuki Shima, Akiyo Yamauchi, Kayo Nakamura, Taeko Sasaki, Tatsuya Kaminishi, Harunori Yoshikawa, Miyuki Sato, Hidetaka Kosako, Tamotsu Yoshimori, Shuhei Nakamura

**Affiliations:** ^1^ Laboratory of Intracellular Membrane Dynamics, Graduate School of Frontier Biosciences Osaka University Osaka Japan; ^2^ Department of Biochemistry Nara Medical University Nara Japan; ^3^ Faculty of Science and Engineering Waseda University Tokyo Japan; ^4^ Center for Autophagy and Anti‐Aging Research, Nara Medical University Nara Japan; ^5^ Laboratory of Molecular Membrane Biology, Institute for Molecular and Cellular Regulation Gunma University Gunma Japan; ^6^ Beyond Cell Reborn Research, Graduate School of Medicine Osaka University Osaka Japan; ^7^ Fujii Memorial Institute of Medical Sciences, Institute of Advanced Medical Sciences Tokushima University Tokushima Japan

## Abstract

Autophagy, a highly conserved cellular degradation process, plays essential roles in various physiological processes including aging. Though autophagy is required for lifespan extension in multiple longevity paradigms, the tissue‐specific roles of autophagy‐related genes (*atgs*) in longevity remain incompletely understood. Here, we investigate the tissue‐specific requirements of *atgs* to promote longevity conferred by germline ablation (called gonadal longevity) using *C. elegans* . Remarkably, we discovered that neuronal or intestinal knockdown of *atg‐18*, but not other *atgs*, specifically abolished gonadal longevity, although knockdown of all tested *atgs* effectively inhibited autophagic activity in these targeted tissues, implying the presence of an autophagy‐independent function of ATG‐18 in gonadal longevity. We demonstrated that germline deficiency triggered significant upregulation of ATG‐18 in neurons and the intestine. From the proteomics analysis and subsequent screening, we found ATG‐18 interacts with PCK‐2, a phosphoenolpyruvate carboxykinase. PCK‐2 is upregulated within the intestine of germline‐deficient animals, but this depends on the non‐autophagic function of ATG‐18. Consistently, we showed that PCK‐2 overexpression mediated longevity required ATG‐18 but not its potential interacting partner to regulate autophagy, ATG‐2. These findings reveal a previously unrecognized autophagy‐independent role for ATG‐18 in regulating lifespan in response to germline signals, expanding our understanding of how this evolutionarily conserved protein coordinates organism‐wide responses to promote longevity.

## Graphical abstract

Neuronal and intestinal ATG‐18 promote longevity conferred by germline removal in *C. elegans* via non‐autophagic functions. In the intestine, ATG‐18 regulates lifespan through its interaction with the gluconeogenic enzyme PCK‐2.

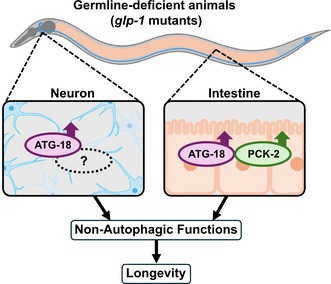

## 1 Introduction

Aging is a complex biological process characterized by the progressive decline of physiological functions over time. Studies using model organisms have revealed that aging, like other biological phenomena, is a regulated process that can be modulated through environmental and genetic interventions. Gonadal longevity, also known as germline longevity, refers to a well‐established phenomenon in *Caenorhabditis elegans* ( *C. elegans* ) where germline removal leads to significantly extended lifespan (Hsin and Kenyon 1999; Arantes‐Oliveira et al. 2002). This longevity paradigm was first discovered through laser ablation of germline precursor cells and later confirmed by genetic mutations that impair germline proliferation, such as *glp‐1* mutants. The absence of germline triggers a complex signaling cascade involving several transcription factors such as DAF‐12, DAF‐16, HLH‐30, PHA‐4, MML‐1, ultimately resulting in metabolic remodeling, activation of various stress resistance pathways, and autophagy (Khodakarami et al. 2015; Olsen and Gill 2017). Notably, this lifespan extension requires the presence of somatic gonad cells, suggesting a delicate balance between germline and somatic signals in regulating organismal aging (Hsin and Kenyon 1999; Yamawaki et al. 2010). Gonadal longevity represents a fascinating example of reproductive trade‐offs in lifespan determination and provides a valuable model for studying the molecular mechanisms connecting reproduction and aging.

Macroautophagy (hereafter autophagy) is a highly conserved degradative pathway where a newly formed membrane, termed isolation membrane or phagophore, elongates and closes to form a double‐membrane vesicle called an autophagosome that engulfs cytoplasmic cargos randomly or selectively and subsequently fuses with lysosomes for degradation and recycling. This process is orchestrated by a set of autophagy‐related (*atg*) genes first identified in yeast and conserved throughout evolution (Mizushima et al. 2011; Nishimura and Tooze 2020). The autophagy process includes several sequential steps such as initiation, nucleation, elongation, and fusion, each requiring specific ATG proteins. The initiation phase involves the ULK1 kinase complex, while nucleation requires the Beclin‐1 complex. The elongation step depends on two ubiquitin‐like conjugation systems and several other proteins, including ATG2 and ATG18, which function in the recruitment of lipids to the growing phagophore membrane. Autophagy operates at basal levels under steady‐state conditions, contributing to cellular metabolic turnover by gradually degrading cytoplasmic components and replacing them with new ones. Furthermore, autophagy is strongly enhanced during starvation or specific stress conditions, breaking down cytoplasmic components into their constituent elements such as amino acids, glucose, and fatty acids to supply essential nutrients for survival (Kuma et al. 2004). Based on these functions, autophagy's physiological roles include cellular metabolic turnover and nutrient acquisition during starvation, making it an essential mechanism for maintaining cellular homeostasis.

Compelling evidence has established activation of autophagy as one of the convergent mechanisms of longevity across diverse organisms (Nakamura and Yoshimori 2018). The decline in autophagic activity is one of the most consistent hallmarks of aging (Nakamura et al. 2019). Remarkably, studies in *C. elegans* demonstrate that multiple independent longevity paradigms—including reduced insulin/IGF‐1 signaling (IIS), dietary restriction (DR), reduced TOR signaling, reduced mitochondrial function, and germline removal—all require activation of autophagy for their beneficial effects on lifespan (Meléndez et al. 2003; Jia and Levine 2007; Tóth et al. 2008; Lapierre et al. 2011; Chen et al. 2019). Moreover, direct activation of autophagy through modulation of key regulators or pharmacological interventions extends lifespan across species (Eisenberg et al. 2009; Harrison et al. 2009; Lapierre et al. 2013; Pyo et al. 2013; Ryu et al. 2016; Fernández et al. 2018). Despite the well‐established importance of autophagy in longevity, a critical knowledge gap persists regarding its tissue‐specific contributions. Most studies have employed systemic genetic manipulations or treatments, leaving the importance of autophagy in individual tissues for organismal longevity largely unexplored. Limited evidence suggests a predominant role for autophagy in neurons, the intestine, and the hypodermis in organismal aging, but this remains incompletely characterized (Nakamura et al. 2019; Chen et al. 2019; Gelino et al. 2016; Minnerly et al. 2017; Kumsta et al. 2019).

Recent studies have revealed that some ATG proteins possess functions beyond their canonical roles in autophagosome formation (Subramani and Malhotra 2013; Shang et al. 2024). These autophagy‐independent functions include Atg5 involvement in the clearance of intracellular microbes, ULK1/ATG1's essential role in ER‐to‐Golgi trafficking for cellular homeostasis, ATG7's functions in modulating p53 activity, and Beclin‐1 participation in endocytic trafficking (Zhao et al. 2008; Lee et al. 2012; Joo et al. 2016; Galluzzi and Green 2019; Tran et al. 2024). These functional redundancies of ATG proteins provide alternative interpretations for genetic studies targeting *atg*s. Therefore, identifying autophagy‐independent functions of ATG proteins is crucial for a comprehensive understanding of the role of autophagy in organismal aging. ATG‐18 is a WD40 repeat‐containing protein that plays a critical role in autophagosome formation by binding to phosphatidylinositol 3‐phosphate (PI3P) to promote phagophore elongation (Lu et al. 2011). Although autophagy‐independent functions of yeast Atg18 and its mammalian homolog WIPI proteins have been reported (Zhu et al. 2024), such non‐autophagic roles have not been documented in *C. elegans* ATG‐18. Therefore, *atg‐18* has been widely employed as a primary target for genetic manipulation to investigate the function of autophagy in various biological processes in *C. elegans* (Minnerly et al. 2017; Chang et al. 2017; Jung et al. 2023; Possik et al. 2023; Franco‐Romero et al. 2024; Metcalf et al. 2024).

In this study, using *C. elegans* , we performed a comprehensive tissue‐specific analysis of autophagy genes in gonadal longevity and discovered a unique role for ATG‐18 in this context. We found that neuronal or intestinal knockdown of *atg‐18*, but not other *atgs*, specifically abolished gonadal longevity. We identified an autophagy‐independent function of ATG‐18 in regulating lifespan through the genetic interaction with PCK‐2, a phosphoenolpyruvate carboxykinase. Our findings reveal the presence of a novel autophagy‐independent function of ATG‐18 essential for gonadal longevity in a tissue‐specific manner, providing additional examples of non‐autophagic ATG function.

## 2 Results

### 2.1 *Atg‐18* Is Essential for the Gonadal Longevity in Both Neurons and the Intestine

**FIGURE 1:**
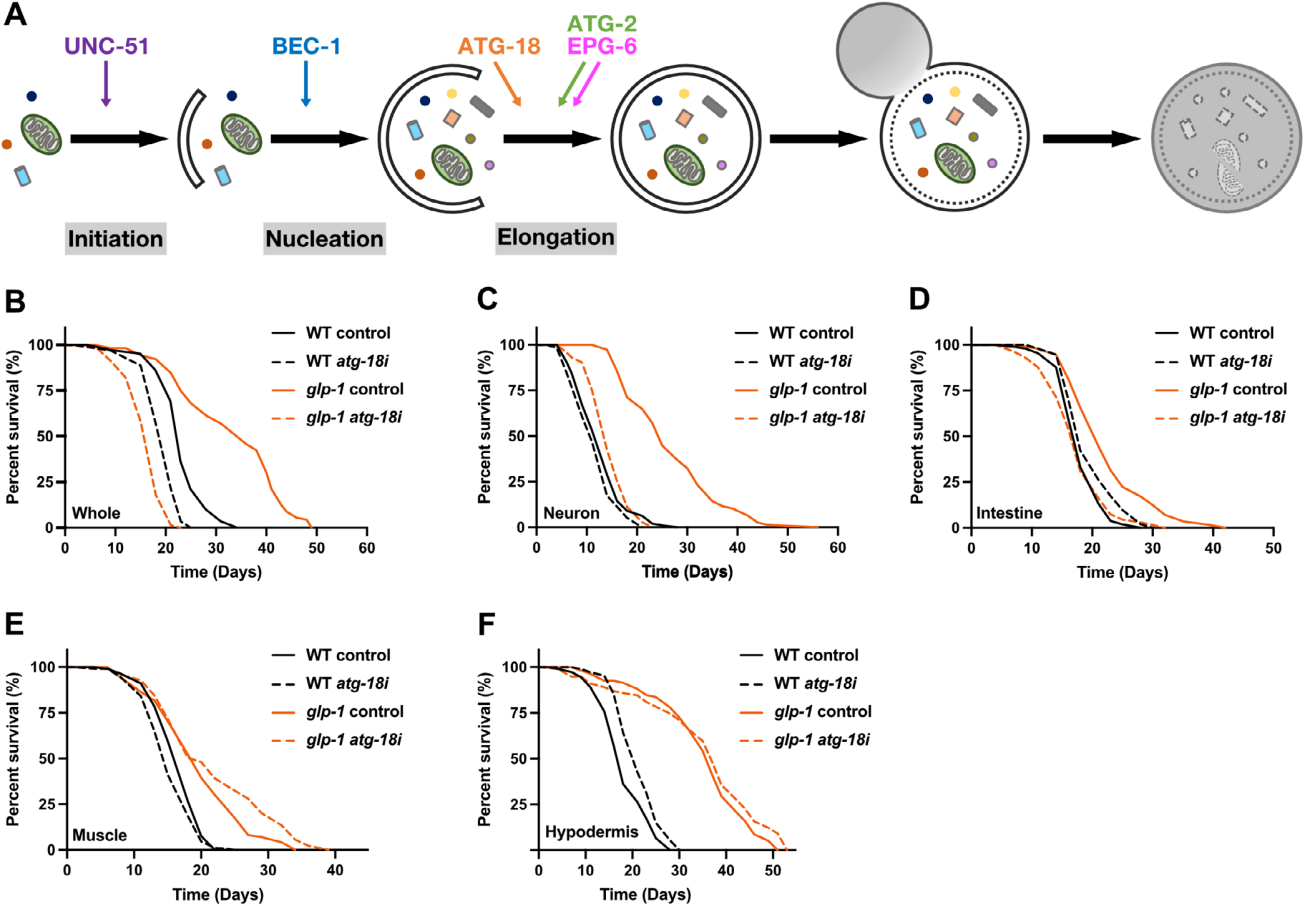
Suppression of *atg‐18* in either neurons or the intestine abolished gonadal longevity. (A) Schematic diagram illustrating the steps involving selected ATGs utilized in this study within the autophagy process. (B) Lifespan analysis of wild‐type (WT) and *glp‐1(e2141)* animals fed bacteria expressing *luciferase* (control) or *atg‐18* dsRNA from adult Day 1. Three biological replicates were performed with 120 worms tested per condition. (C–F) Lifespan analysis of wild‐type (WT) and *glp‐1(e2141)* animals capable of tissue‐specific RNAi (C: Neuron (TU3401), D: Intestine (VP303), E: Muscle (NR350), F: Hypodermis (NR222)) fed bacteria expressing *luciferase* (control) or *atg‐18* dsRNA from adult Day 1. Panels C and D represent three biological replicates with 120 worms tested per condition. Panels E and F represent two biological replicates with 120 worms tested per condition.

To confirm previous findings that autophagy is required for gonadal longevity and to validate the efficacy of our RNA interference (RNAi) approach, we first conducted lifespan analysis in *glp‐1* mutants subjected to systemic RNAi of *atg‐18* which is essential for autophagosome elongation (Figure 1A). Consistent with the previous report (Lapierre et al. 2011), we observed that whole‐body suppression of *atg‐18* from adult onward significantly abolished the longevity of *glp‐1* mutants (Figure 1B). Next, to elucidate the tissue‐specific contribution of *atg‐18* in gonadal longevity, we employed the tissue‐specific RNAi approach as previously described in our recent work (Shioda et al. 2023). These strains allow for selective knockdown of gene expression in specific tissues including neurons, intestine, muscle, and hypodermis of germline‐deficient animals (Espelt et al. 2005; Qadota et al. 2007; Calixto et al. 2010). Of note, the conventional neuronal RNAi strain (TU3401) has been reported to exhibit RNAi leakage to non‐neuronal tissues (Gahlot and Singh 2024). To address this concern, we utilized an additional, more specific neuronal RNAi strain (Yang et al. 2024), enabling us to assess the role of *atg‐18* in neurons. Lifespan analysis revealed that neuronal or intestinal suppression of *atg‐18* specifically abolished the longevity conferred by germline deficiency (Figures 1C,D, and S1). In contrast, knockdown of *atg‐18* in muscle or hypodermal tissue had no effect on the *glp‐1* longevity (Figure 1E,F). Importantly, none of these tissue‐specific *atg‐18* RNAi affected the lifespan of wild‐type (WT) animals, suggesting that *atg‐18* function in neurons and the intestine is specifically required for gonadal longevity in *C. elegans* .

To further clarify the tissue‐specific requirement of *atg‐18* in gonadal longevity, we conducted rescue experiments. We generated transgenic animals expressing ATG‐18::GFP driven by its endogenous promoter to verify functionality, or driven by tissue‐specific promoters (*rab‐3* promoter for neurons; *ges‐1* promoter for the intestine) to test tissue‐specific requirements (Figure S2A,B). ATG‐18::GFP driven by its endogenous promoter fully rescued, and even further extended, the shortened lifespan of *glp‐1; atg‐18* mutants, confirming the GFP‐tagged construct is functional (Figure S2C). However, tissue‐specific expression of ATG‐18::GFP in neurons or the intestine alone, or even in both tissues simultaneously, failed to restore longevity (Figure S2C). These findings suggest that rescue of ATG‐18 in a single tissue is insufficient for the full manifestation of gonadal longevity in *C. elegans* .

### 2.2 Autophagic Inhibition in Any Single Tissue Does Not Affect Gonadal Longevity

**FIGURE 2:**
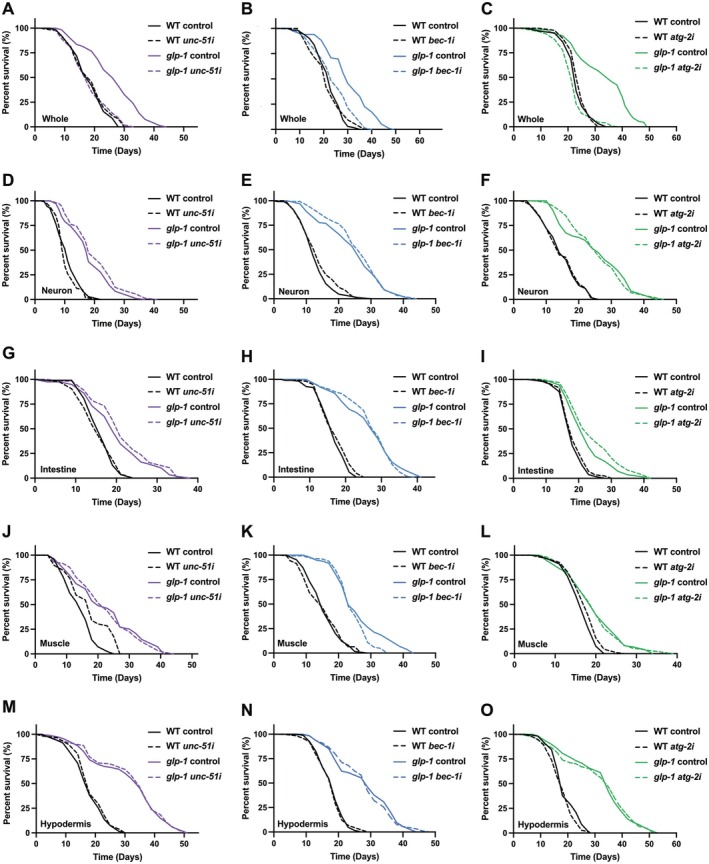
Tissue‐specific suppression of *atgs* other than *atg‐18* has no effect on *glp‐1* longevity. (A–C) Lifespan analysis of wild‐type (WT) and *glp‐1(e2141)* animals fed bacteria expressing *luciferase* (control), (A) *unc‐51*, (B) *bec‐1*, or (C) *atg‐2* dsRNA from adult day 1. Three biological replicates were performed with 120 worms tested per condition. (D–F) Lifespan analysis of wild‐type (WT) and *glp‐1(e2141)* animals capable of neuron‐specific RNAi fed bacteria expressing *luciferase* (control), (D) *unc‐51*, (E) *bec‐1*, or (F) *atg‐2* dsRNA from adult day 1. Two biological replicates were performed with 120 worms tested per condition. (G–I) Lifespan analysis of wild‐type (WT) and *glp‐1(e2141)* animals capable of intestine‐specific RNAi fed bacteria expressing *luciferase* (control), (G) *unc‐51*, (H) *bec‐1*, or (I) *atg‐2* dsRNA from adult day 1. Two biological replicates were performed with 120 worms tested per condition. (J–L) Lifespan analysis of wild‐type (WT) and *glp‐1(e2141)* animals capable of muscle‐specific RNAi fed bacteria expressing *luciferase* (control), (J) *unc‐51*, (K) *bec‐1*, or (L) *atg‐2* dsRNA from adult day 1. Two biological replicates were performed with 120 worms tested per condition. (M–O) Lifespan analysis of wild‐type (WT) and *glp‐1(e2141)* animals capable of hypodermis‐specific RNAi fed bacteria expressing *luciferase* (control), (M) *unc‐51*, (N) *bec‐1*, or (O) *atg‐2* dsRNA from adult day 1. Two biological replicates were performed with 120 worms tested per condition.

Following our finding that *atg‐18* plays a tissue‐specific role in gonadal longevity, we next investigated whether inhibition of other *atgs* would produce a similar effect. To comprehensively evaluate the autophagy pathway, we selected genes representing key sequential steps in autophagosome formation: *unc‐51* (initiation), *bec‐1* (nucleation), and *atg‐2* (elongation); *C. elegans* homologs of ULK1, Beclin‐1, and ATG2 (Figure 1A). First, we confirmed that systemic RNAi of these genes from adult onward abolished the extended lifespan of *glp‐1* mutants. As expected, suppression of *unc‐51*, *bec‐1*, or *atg‐2* significantly reduced the *glp‐1* longevity (Figure 2A–C), consistent with the established requirement for autophagy in gonadal longevity. Surprisingly, unlike our observations with *atg‐18*, knockdown of these other *atgs* in any single tissue was insufficient to suppress the *glp‐1* longevity (Figure 2D–O). The differential effects observed between tissue‐specific RNAi of *atg‐18* versus other *atgs* suggest a non‐canonical role for ATG‐18 in gonadal longevity. Specifically, the ability of neuronal or intestinal *atg‐18* RNAi, but not RNAi of other *atgs*, to suppress the *glp‐1* longevity suggests that neuronal and intestinal ATG‐18 may possess an autophagy‐independent function that is essential for gonadal longevity.

### 2.3 Tissue‐Specific Knockdown of *Atgs* Certainly Leads to Impaired Autophagy in the Affected Tissue

**FIGURE 3:**
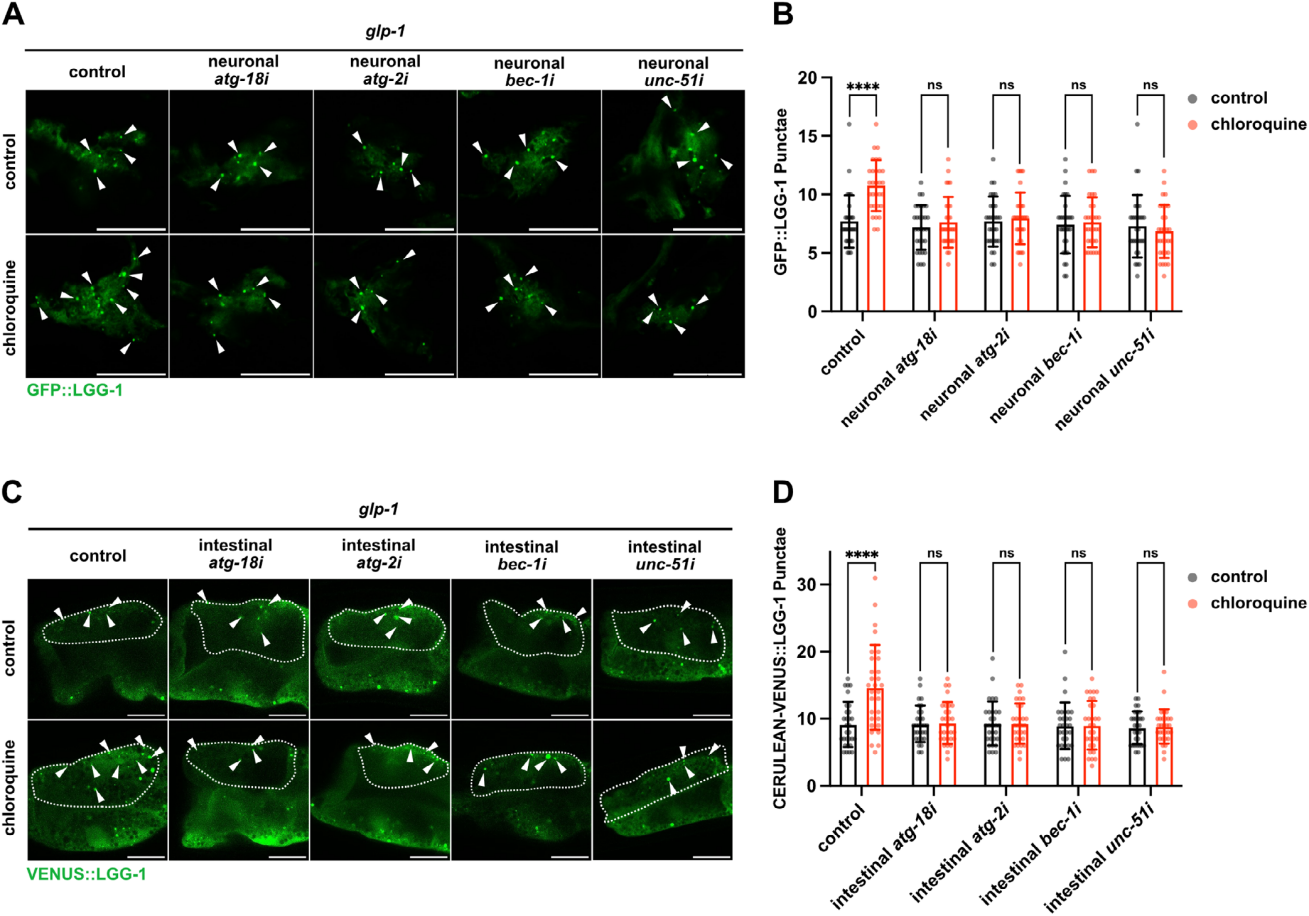
The autophagy flux in its target tissue is impaired by neuronal‐ or intestinal‐specific RNAi of *atgs*. (A) Representative fluorescent images of GFP::LGG‐1 puncta in nerve‐ring neurons of *glp‐1(e2141)* animals treated with or without 5 mM chloroquine on adult day 1. Knockdown was conducted from egg onward. Each arrow indicates GFP::LGG‐1 puncta. (B) Quantification of GFP::LGG‐1 puncta in neurons shown in (A). Values represent mean ± SD (*n* = 30). *p* values (ns > 0.05, ****p < 0.0001) were determined by two‐way ANOVA with Tukey's test. (C) Representative fluorescent images of VENUS::LGG‐1 puncta in intestinal cells of *glp‐1(e2141)* animals treated with or without 5 mM chloroquine on adult day 1. Knockdown was conducted from egg onward. Each arrow indicates VENUS::LGG‐1 puncta. Dash lines indicate single intestinal cells. (D) Quantification of VENUS::LGG‐1 puncta in the intestinal cell shown in (C). Values represent mean ± SD (*n* = 30). *p* values (ns > 0.05, ****p < 0.0001) were determined by two‐way ANOVA with Tukey's test.

One possible explanation for the inability of tissue‐specific suppression of *atgs* except for *atg‐18* to abolish gonadal longevity could be attributed to insufficient knockdown efficiency that did not effectively impair autophagic activity. To exclude this possibility, we assessed the autophagic activity in tissues affected by tissue‐specific suppression of each *atg*. To monitor the activity of autophagy, we established *glp‐1* mutants expressing the autophagosome reporter GFP::LGG‐1/ATG8 with tissue‐specific RNAi capabilities and measured autophagy flux using chloroquine treatment, which inhibits lysosomal function to prevent autophagy flux. First, we confirmed enhanced autophagy flux in neurons of *glp‐1* mutants under control conditions, as chloroquine treatment significantly increased GFP::LGG‐1 puncta compared to untreated animals (Figure 3A,B). On the other hand, in *glp‐1* mutants with neuron‐specific knockdown of *atg‐18*, *unc‐51*, *bec‐1*, or *atg‐2*, the numbers of GFP::LGG‐1 puncta were not significantly altered following chloroquine treatment. This indicates that neuron‐specific knockdown of any of these *atgs* effectively impaired autophagy flux in neurons of germline‐deficient animals. Similarly, we confirmed that intestinal RNAi of each *atg* successfully disrupted autophagy flux in the intestinal cells (Figure 3C,D). Muscle‐specific knockdown of each *atg* also effectively impaired autophagy flux in muscle tissue of germline‐deficient animals (Figure S3A,B). Although we attempted to assess autophagy flux in the hypodermis, the fluorescence signal from the hypodermis‐specific autophagy reporter (DLM6) was insufficient in our hands, precluding reliable quantification. Collectively, these findings suggest that the differential phenotypic outcomes between *atg‐18* RNAi and other *atgs* RNAi in gonadal longevity are not attributable to differences in knockdown efficiency. These findings further support the hypothesis that ATG‐18 possesses autophagy‐independent functions essential for gonadal longevity in both neurons and the intestine.

To provide further evidence for this autophagy‐independent role, we examined the subcellular localization of ATG‐18 relative to autophagosomes. We co‐expressed ATG‐18::GFP with mCherry::LGG‐1 in *glp‐1* mutants and found that ATG‐18 showed minimal colocalization with LGG‐1 puncta in both neurons and the intestine at adult day 1 (Figure S4A,B), suggesting that ATG‐18 largely resides outside of autophagosomes. The functionality of the ATG‐18::GFP reporter was validated by observing clear colocalization with LGG‐1 during larval stages (Figure S4C). These observations provide additional evidence that ATG‐18 functions independently of canonical autophagy in germline‐deficient animals.

### 2.4 Differential Contribution of PROPPIN Family to Gonadal Longevity in *C. elegans*

*C. elegans* possesses another conserved PROPPIN family member, EPG‐6 (mammalian homologs: WIPI3/4), in addition to ATG‐18 (mammalian homologs: WIPI1/2). ATG‐18 and EPG‐6 share structural similarities but have been shown to function at different steps of the autophagy process (Figure 1A; Lu et al. 2011). Interestingly, EPG‐6 has been reported to have non‐autophagic functions that influence the lifespan of WT animals (Takacs et al. 2019). Given the autophagy‐independent role of ATG‐18 in gonadal longevity, we sought to determine whether EPG‐6 might exhibit similar properties in this context. Systemic suppression of *epg‐6* completely abolished the extended lifespan of *glp‐1* mutants (Figure S5A), whereas specific suppression of *epg‐6* in neurons, intestine, muscle, or hypodermis did not affect the *glp‐1* longevity (Figure S5B–E). Furthermore, we found that neuron‐, intestine‐, or muscle‐specific suppression of *epg‐6* effectively inhibited autophagy flux in the target tissues (Figure S5F–K). These findings suggest that the autophagy‐independent function of ATG‐18 in lifespan regulation is not widely conserved across the PROPPIN family, highlighting the unique role of ATG‐18 in regulating lifespan conferred by germline deficiency.

### 2.5 Germline Deficiency Increases ATG‐18 Expression in Neurons and the Intestine

**FIGURE 4:**
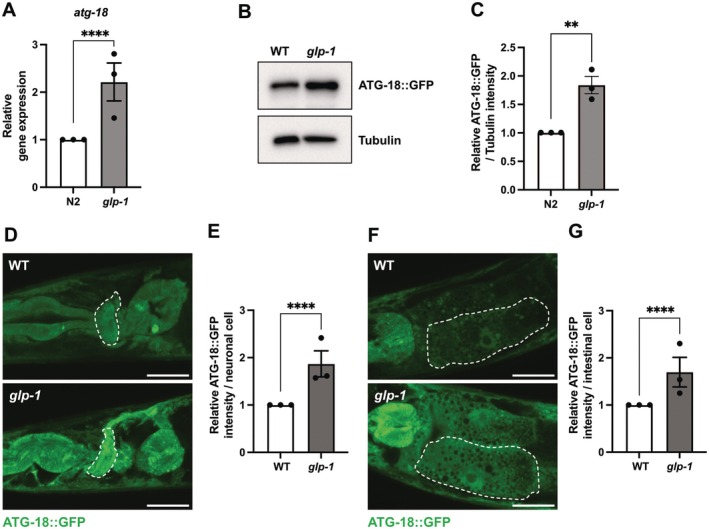
ATG‐18 is up‐regulated in both neurons and the intestine of germline‐deficient animals. (A) qRT‐PCR analysis of *atg‐18* expression in wild‐type (WT) and *glp‐1(e2141)* animals on adult day 1. Values represent mean ± SD from three biological replicates. *p* value (****p < 0.0001) was determined by *t*‐test. (B) Representative western blots of ATG‐18::GFP in wild‐type (WT) and *glp‐1(e2141)* animals on adult day 1. (C) Quantification of ATG‐18::GFP expression shown in (B). Values represent mean ± SD from three biological replicates. *p* value (**p < 0.01) was determined by *t*‐test. (D) Representative fluorescent images of ATG‐18::GFP transgenic worms in nerve‐ring neurons of wild‐type (WT) and *glp‐1(e2141)* animals on adult day 1. Scale bars, 20 μm. Dash lines indicate the nerve‐ring neuron. (E) Quantification of ATG‐18::GFP in neurons shown in (D). Values represent mean ± SD from three biological replicates (10 worms each). *p* value (****p < 0.0001) was determined by *t*‐test. (F) Representative fluorescent images of ATG‐18::GFP transgenic worms in the intestinal cell of wild‐type (WT) and *glp‐1(e2141)* animals on adult Day 1. Scale bars, 20 μm. Dash lines indicate single intestinal cells. (G) Quantification of ATG‐18::GFP in the intestinal cells shown in (F). Values represent mean ± SD from three biological replicates (10 worms each). *p* value (****p < 0.0001) was determined by *t*‐test.

We next investigated whether germline deficiency affects ATG‐18 expression. qRT‐PCR analysis revealed that the *atg‐18* transcript abundance was significantly elevated in *glp‐1* mutants at the whole‐body level (Figure 4A). We then crossed *glp‐1* mutants with ATG‐18::GFP transgenic worms and analyzed the expression levels of ATG‐18::GFP. Consistent with the transcriptional changes, we observed increased ATG‐18::GFP expression induced by germline deficiency (Figure 4B,C). To determine the tissue‐specific changes in ATG‐18 expression, we conducted confocal microscopy analysis of ATG‐18::GFP in neurons and the intestine. Interestingly, we found that the fluorescence intensity of ATG‐18::GFP was significantly increased in these tissues of *glp‐1* mutants compared with WT animals (Figure 4D–G). The upregulation of ATG‐18 in the tissues required for lifespan extension further emphasizes the importance of ATG‐18 in neurons and the intestine for gonadal longevity.

### 2.6 Proteomics Analysis Identifies Potential ATG‐18 Interactors in the Context of Gonadal Longevity

**FIGURE 5:**
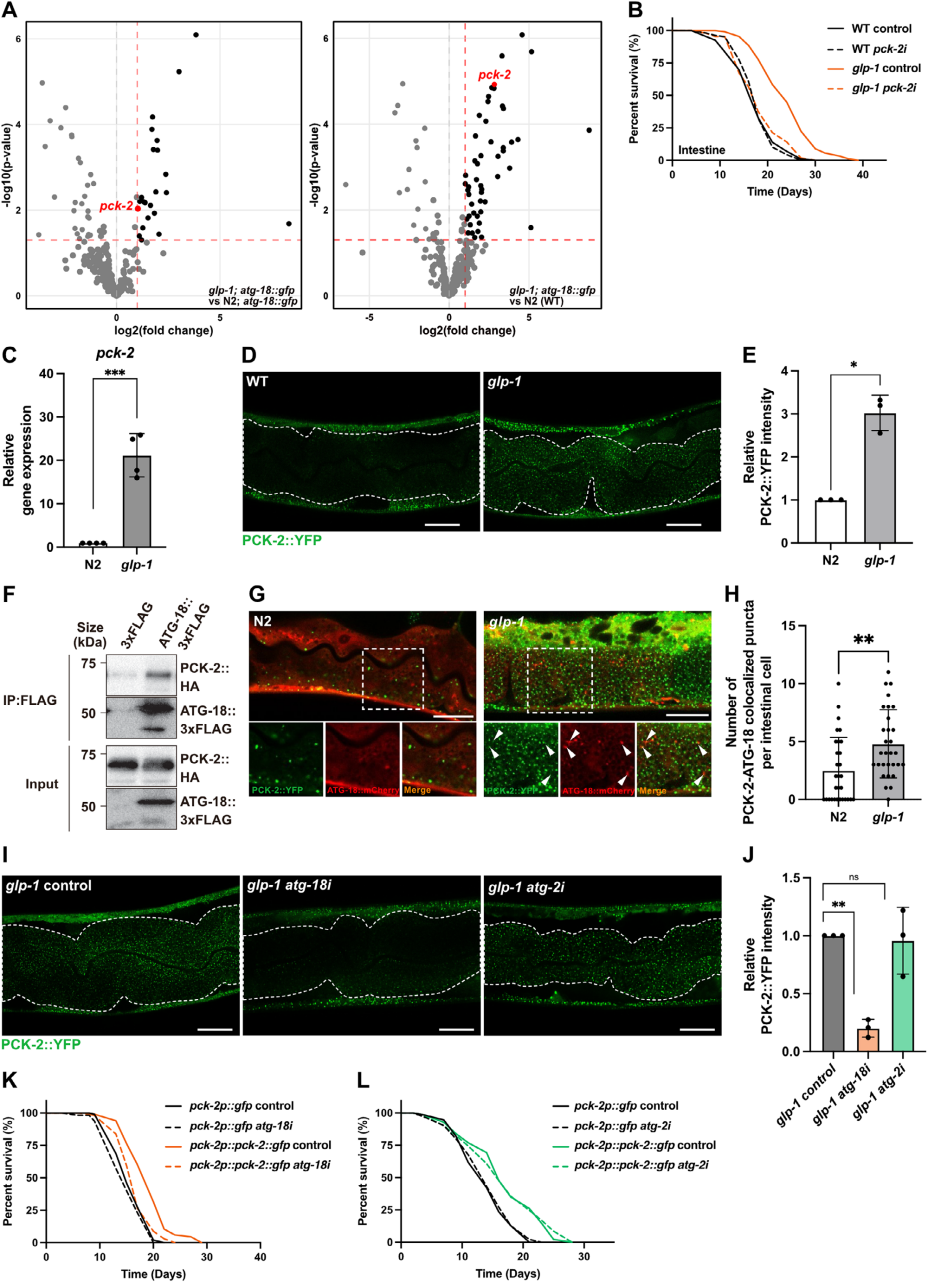
PCK‐2 mediates autophagy‐independent lifespan extension by ATG‐18 in *glp‐1* mutants. (A) Volcano plots of ATG‐18::GFP interacting proteins. Log2 fold changes are plotted against –log10 *p* value for *glp‐1; atg‐18::gfp* vs. N2; *atg‐18::gfp* (left panel) and *glp‐1; atg‐18::gfp* vs. N2 (right panel) comparisons. Proteins were considered significantly differentially expressed if *p* < 0.05 and log2 fold change > 1. The protein of interest (PCK‐2) is colored red, other significant proteins are colored black, and non‐significant proteins are colored gray. A red dashed line indicates the significance thresholds. (B) Lifespan analysis of wild‐type (WT) and *glp‐1(e2141)* animals capable of intestine‐specific RNAi fed bacteria expressing *luciferase* (control) or *pck‐2* dsRNA from egg onward. Three biological replicates were performed with 120 worms tested per condition. (C) qRT‐PCR analysis of *pck‐2* expression in wild‐type (WT) and *glp‐1(e2141)* animals on adult Day 1. Values represent mean ± SD from three biological replicates. *p* value (***p < 0.001) was determined by *t*‐test. (D) Representative fluorescent images of PCK‐2::YFP transgenic worms in the intestine of wild‐type (WT) and *glp‐1(e2141)* animals on adult day 1. Scale bars, 20 μm. Dash lines indicate intestinal cells. (E) Quantification of PCK‐2::YFP in the intestines shown in (D). Values represent mean ± SD from three biological replicates (10 worms each). *p* value (*p < 0.05) was determined by *t*‐test. (F) Co‐immunoprecipitation of ATG‐18 and PCK‐2. HEK293 cells were co‐transfected with PCK‐2::HA and ATG‐18::3xFLAG. Immunoprecipitation with anti‐FLAG antibody pulled down PCK‐2::HA, confirming physical interaction between ATG‐18 and PCK‐2. (G) Representative images showing colocalization of PCK‐2::YFP and ATG‐18::mCherry in the intestinal cell of N2 and *glp‐1(e2141)* animals. Scale bars, 20 μm. Each arrow indicates a punctum where PCK‐2 and ATG‐18 colocalize. (H) Quantification of PCK‐2‐ATG‐18 colocalized puncta per intestinal cell shown in (G). Values represent mean ± SD (*n* = 30). *p* values (**p < 0.01) were determined by *t‐test*. (I) Representative fluorescent images of PCK‐2::YFP transgenic worms in the intestine of *glp‐1(e2141)* animals fed bacteria expressing *luciferase* (control), *atg‐18*, or *atg‐2* dsRNA from adult Day 1. Scale bars, 20 μm. Dash lines indicate intestinal cells. (J) Quantification of PCK‐2::YFP in the intestines shown in (I). Values represent mean ± SD from three biological replicates (10 worms each). *p* value (**p < 0.01) was determined by *t*‐test. (K and L) Lifespan analysis of *pck‐2p::gfp* expressing animals and *pck‐2p::pck‐2::gfp* expressing animals fed bacteria expressing (K) *atg‐18*, or (L) *atg‐2* dsRNA from adult Day 1. Three biological replicates were performed with 120 worms tested per condition.

In yeast, Atg18 interacts with Atg2 and PI3P, mediating lipid transport by tethering membranes and proteins (Obara et al. 2008; Watanabe et al. 2012). If *C. elegans* ATG‐18 also possesses such functions, we hypothesized that ATG‐18 does not function as a single molecule in lifespan extension but rather exerts its function through interaction with certain factors. To explore the unknown function of ATG‐18, we aimed to obtain an interaction profile of ATG‐18 in the context of gonadal longevity. For this purpose, we conducted interactome analysis for ATG‐18::GFP both in WT and *glp‐1* animals. Our proteomics analysis identified 35 candidates that showed significantly enhanced interaction with ATG‐18::GFP in *glp‐1* mutants compared to WT (Figures 5A and S6A). Notably, components of the retromer complex (VPS‐26, VPS‐29, VPS‐35), which have been recently implicated in the non‐canonical functions of Atg18 in yeast, were undetected in our list of ATG‐18 interactors in *C. elegans* (Courtellemont et al. 2022). To validate the effect of these candidates in aging, we generated *glp‐1; rrf‐3* double mutants with enhanced RNAi sensitivity throughout the body including neurons (Simmer et al. 2002) and conducted survival screening to identify factors relevant to gonadal longevity (Figure S6B). For our screening, we selected candidate factors according to the following criteria based on the proteomics data: (i) relative survival rate < 0.6 at day 25, (ii) log2 fold‐change > |1|, *p*‐value < 0.05 for *glp‐1; atg‐18::gfp* vs. *N2; atg‐18::gfp* comparison, (iii) log2 fold‐change > 1 for *glp‐1; atg‐18::gfp* vs. *N2; atg‐18::gfp* comparison, (iv) log2 fold‐change > |1|, *p*‐value < 0.05 for *glp‐1; atg‐18::gfp* vs. *N2* comparison, and (v) log2 fold‐change > |1|, *p*‐value < 0.05 for *N2; atg‐18::gfp* vs. *N2* comparison. Among the 35 candidates, two factors, *pck‐2* and *tbh‐1*, met all these stringent criteria. To further validate these candidates, we performed full‐scale lifespan analysis using *glp‐1; rrf‐3* mutants. While *tbh‐1* knockdown showed no reproducible effect on gonadal longevity, *pck‐2* RNAi significantly abolished the extended lifespan of *glp‐1* mutants (Figure S7A,B). To determine the tissue‐specific requirement of these factors, we next examined their roles in neurons and the intestine using tissue‐specific RNAi. Neuronal suppression of either *pck‐2* or *tbh‐1* had no effect on the *glp‐1* longevity (Figure S7C,D). In contrast, intestinal knockdown of *pck‐2* abolished gonadal longevity, while intestinal *tbh‐1* did not (Figures 5B and S7E). These findings identify PCK‐2 as a critical factor that collaborates with ATG‐18 in the autophagy‐independent regulation of gonadal longevity, particularly within the intestinal tissue.

### 2.7 Autophagy‐Independent Lifespan Regulation by ATG‐18 Is Mediated by PCK‐2

PCK‐2 encodes phosphoenolpyruvate carboxykinase, a key enzyme in gluconeogenesis that catalyzes the conversion of oxaloacetate to phosphoenolpyruvate and has been implicated in metabolic regulation and longevity (Onken et al. 2020). Having identified PCK‐2 as a critical collaborator of ATG‐18 in gonadal longevity, we next investigated the relationship between ATG‐18 and PCK‐2 in germline‐deficient animals.

We first examined the expression of PCK‐2 in germline‐deficient animals. qRT‐PCR analysis revealed that *pck‐2* transcript levels were significantly elevated in *glp‐1* mutants compared to WT animals (Figure 5C). To examine this upregulation at the protein level, we crossed *glp‐1* mutants with worms in which YFP was knocked in at the C‐terminus of PCK‐2. The PCK‐2::YFP fluorescence was significantly enhanced in *glp‐1* mutants both in the whole body and the intestine (Figures 5D,E, and S8A,B), confirming increased PCK‐2 expression at the protein level in germline‐deficient animals.

We next sought to validate the physical interaction between ATG‐18 and PCK‐2 identified by our proteomics analysis. We initially attempted to perform co‐immunoprecipitation in *C. elegans* , but double transgenic animals co‐expressing tagged ATG‐18 and PCK‐2 in the *glp‐1* background exhibited unexpectedly reduced fertility, precluding collection of sufficient material. We therefore expressed codon‐optimized *C. elegans* ATG‐18::3xFLAG and PCK‐2::HA in HEK293 cells to validate this interaction. Co‐immunoprecipitation with anti‐FLAG antibody successfully pulled down PCK‐2::HA, confirming the physical interaction between ATG‐18 and PCK‐2 (Figure 5F). Furthermore, we generated animals co‐expressing PCK‐2::YFP and ATG‐18::mCherry and examined their colocalization in the intestine. In *glp‐1* mutants, PCK‐2 and ATG‐18 showed clear colocalization, which was enhanced compared to WT animals (Figure 5G,H). To determine whether this interaction occurs independently of autophagosomes, we expressed PCK‐2::YFP with mCherry::LGG‐1 in *glp‐1* mutants. PCK‐2 showed no colocalization with LGG‐1 puncta in the intestine (Figure S8D), indicating that PCK‐2 localizes entirely independently of autophagosomes. These results imply that ATG‐18 associates with PCK‐2 outside of autophagosomes.

We also examined whether ATG‐18 and PCK‐2 regulate each other's expression. While intestinal ATG‐18::GFP fluorescence was not affected by pck‐2 knockdown in glp‐1 mutants (Figure S8E,F), pck‐2 knockdown unexpectedly altered whole‐body atg‐18 mRNA levels, suggesting the existence of a compensatory mechanism (Figure S8G). In contrast, *atg‐18* knockdown resulted in a decrease in intestinal PCK‐2::YFP fluorescence (Figure 5I,J). Importantly, this effect was specific to *atg‐18*, as *atg‐2* suppression did not alter PCK‐2 expression, further supporting an autophagy‐independent role for ATG‐18. Since *atg‐18* knockdown did not affect *pck‐2* transcript levels (Figure S8H), ATG‐18 regulates PCK‐2 at the post‐transcriptional level.

Finally, we tested whether PCK‐2‐mediated longevity requires ATG‐18. PCK‐2 overexpression significantly extended lifespan in WT animals (Onken et al. 2020). However, this longevity conferred by PCK‐2 overexpression was completely abolished by *atg‐18* suppression, whereas *atg‐2* was not required (Figure 5K,L). These findings establish a novel role for ATG‐18 in gonadal longevity through PCK‐2‐mediated autophagy‐independent function.

## 3 Discussion

Our studies on the tissue‐specific roles of ATG proteins in gonadal longevity have revealed unexpected insights into the autophagy‐independent function of ATG‐18. Through tissue‐specific knockdown experiments, we demonstrated that neuronal or intestinal inhibition of *atg‐18*, but not other *atgs*, specifically suppressed the extended lifespan of *glp‐1* mutants. This finding was particularly striking because all tested *atgs* effectively inhibited autophagy in their respective tissues, yet only *atg‐18* affected longevity. The subsequent proteomic analysis identified novel proteins that could preferentially interact with ATG‐18 in germline‐deficient animals. Among these interactors, we focused on PCK‐2, demonstrating that ATG‐18 affects PCK‐2 expression in the intestine. PCK‐2 overexpression‐mediated lifespan extension required ATG‐18 but not ATG‐2, implying longevity was independent of general autophagy function. These findings establish a novel regulatory role linking ATG‐18 to lifespan regulation through mechanisms distinct from its canonical role in autophagy.

Our discovery of the autophagy‐independent roles of ATG‐18 raises important questions about the mechanisms underlying non‐canonical functions of ATG proteins. Notably, autophagy‐independent functions of ATG‐18/WIPI family proteins have been reported across species. In yeast, Atg18 forms a complex with retromer subunits (termed CROP complex) to mediate membrane fission in the endo‐lysosomal system, independently of its canonical autophagy function (Courtellemont et al. 2022). In mammals, loss of WIPI4 causes ferroptosis by increasing ATG2‐mediated phosphatidylserine transport to mitochondria at ER‐mitochondria contact sites (Zhu et al. 2024). Our finding that ATG‐18 regulates gonadal longevity through PCK‐2 adds to this growing list of non‐canonical ATG‐18/WIPI functions. Similarly, other ATG proteins also possess non‐canonical functions in a context‐dependent manner. Recent findings have demonstrated that ATG‐16.2 possesses autophagy‐independent functions in the regulation of exopher formation and neuronal proteostasis in *C. elegans* (Yang et al. 2024). Furthermore, the unique function of VPS‐34, which is conserved from nematodes to mice and influences motor aging, has also been suggested to be unrelated to autophagy (Hu et al. 2023). These reports on the non‐canonical functions of several ATGs, including this study, emphasize the importance of using multiple gene targets and methodologies to assess the involvement of autophagy in specific biological processes. The approaches that rely solely on single *atg* manipulation or one type of autophagy marker may fail to distinguish between canonical autophagy effects and non‐canonical functions of ATG proteins. Future studies should utilize a combination of methodologies, including flux assays with lysosomal inhibitors, tandem‐tagged autophagy reporters, and genetic approaches targeting two or more *atgs* involved in different steps of the pathways as recommended by recent guidelines for autophagy monitoring (Klionsky et al. 2021). Such multifaceted approaches would provide a more comprehensive evaluation of autophagy status and help identify potential autophagy‐independent functions of other ATG proteins.

An intriguing aspect of this study is that tissue‐specific suppression of most *atgs* did not affect gonadal longevity, despite effective autophagy inhibition in the target tissues. Several hypotheses might explain this observation. First, autophagy might be required at the whole‐body level, with a certain threshold of global autophagic activity necessary for lifespan extension. In this model, inhibition in any single tissue would be insufficient to reduce autophagic activity below the critical threshold, as the remaining tissues could maintain adequate organismal autophagy function. Alternatively, autophagy might be required in specific combinations of tissues to promote longevity. Under this hypothesis, simultaneous inhibition of autophagy in multiple major tissues would be necessary to observe significant effects on lifespan. While our study examined four major tissues of *C. elegans* , we cannot exclude the possibility that autophagy in other specific tissues not targeted in this study, such as the pharynx or excretory system, might be particularly critical for gonadal longevity. Importantly, the tissue‐specific requirements for autophagy likely vary depending on the longevity pathways being activated. Although autophagy is recognized as a convergent mechanism in multiple longevity pathways (Nakamura and Yoshimori 2018; Tóth et al. 2008), it is intriguing to integrate previous reports and this study to see differences in the tissue‐specific autophagy requirements among these pathways. For instance, previous studies demonstrated that intestinal autophagy is necessary for DR‐mediated longevity, contributing to the maintenance of intestinal homeostasis and preventing decline in locomotion associated with aging (Gelino et al. 2016). In contrast, autophagy in the hypodermis contributes to cold‐induced longevity by delaying the reduction of collagen associated with aging (Chen et al. 2019). These studies collectively suggest that autophagy not only exhibits differential tissue requirements that vary according to the longevity pathways involved, but that the downstream mechanisms through which autophagy influences lifespan extension may also differ substantially between distinct longevity paradigms. Beyond these investigations, knockdown studies have suggested that intestinal autophagy is also essential for IIS‐mediated longevity and gonadal longevity (Chang et al. 2017). Complementary rescue experiments have indicated tissue‐specific requirements for autophagy in diverse longevity paradigms: neuronal or intestinal autophagy appears critical for DR‐mediated longevity, while neuronal, intestinal, or hypodermal autophagy are necessary for IIS‐mediated longevity (Minnerly et al. 2017). However, these findings warrant cautious interpretation, as they exclusively targeted *atg‐18* for validation. There exists substantial probability that these studies inadvertently conflated canonical autophagy with autophagy‐independent functions of *atg‐18*. Future studies employing combinatorial approaches, such as simultaneous tissue‐specific knockdown in multiple tissues, could help resolve these questions and provide a more comprehensive understanding of autophagy's tissue‐specific contributions to longevity.

Our findings establish a functional connection between ATG‐18 and PCK‐2 in lifespan regulation, although the precise molecular mechanism requires further investigation. PCK‐2, a phosphoenolpyruvate carboxykinase, catalyzes a rate‐limiting step in gluconeogenesis and has been implicated in metabolic adaptation during dietary restriction. Previous studies have demonstrated that *pck‐2* transcripts increase in *eat‐2* mutants later in life and that upregulation of *pck‐2* is essential for DR‐mediated longevity (Onken et al. 2020), consistent with our observations. Intriguingly, PCK‐2 has also been shown to influence fat metabolism and mitochondrial functions (Franckhauser et al. 2002, 2006; Olswang et al. 2002; Burgess et al. 2004), processes that are known to be altered in germline‐deficient animals (O'Rourke et al. 2009; Hansen et al. 2013; Wei and Kenyon 2016; Chaturbedi and Lee 2025; Held et al. 2024). The post‐transcriptional regulation of PCK‐2 by ATG‐18 suggests several possibilities. ATG‐18 might interact with PCK‐2 to stabilize the protein, as has been observed for other WD40 repeat‐containing proteins and their binding partners (Dahlberg and Juo 2014). Alternatively, ATG‐18 could influence PCK‐2 mRNA translation efficiency, perhaps by interacting with RNA‐binding proteins or translational machinery components. The involvement of ATG‐18's phosphoinositide‐binding capability is particularly intriguing. Through its WD40 domain, ATG‐18 binds to PI3P and PI(3,5)P2, which are important for membrane dynamics and signaling (Baskaran et al. 2012; Tamura et al. 2013). This binding capacity might enable ATG‐18 to function as a scaffold protein, bringing together factors involved in PCK‐2 regulation at specific membrane interfaces. Metabolically, enhanced gluconeogenesis driven by increased PCK‐2 activity could provide metabolic flexibility during periods of stress or reduced nutrient availability, which might be important in germline‐deficient animals undergoing significant physiological remodeling.

Although ATG‐18 contributes to gonadal longevity in both neurons and the intestine, our proteomic analysis primarily identified intestinal interactors. This observation suggests that ATG‐18 might employ different mechanisms in these two tissue types. The relative paucity of neuronal interaction factors may be due to technical limitations, such as the low abundance of neuronal cells, or it may reflect true biological differences in ATG‐18 function across tissues. In neurons, which are specialized for information processing and transmission, ATG‐18 could influence processes in neuroendocrine signaling pathways that are critical for lifespan regulation in *C. elegans* . Specific neuroendocrine signals have been implicated in communicating germline status to peripheral tissues (Shioda et al. 2023; Singh et al. 2022). A recent study demonstrated that neuronal ATG‐18 functions in insulin‐like signaling pathways to regulate lipid metabolism in intestinal cells through neuroendocrine mechanisms (Jia et al. 2019). This work provides compelling evidence for a tissue‐specific role of ATG‐18 in inter‐tissue communication that affects longevity, consistent with our observations that neuronal ATG‐18 is essential for gonadal longevity. Future studies focusing specifically on neuronal ATG‐18 using neuron‐specific expression systems or more sensitive detection methods could reveal additional mechanisms through which ATG‐18 contributes to longevity in neurons. Understanding these tissue‐specific functions and their coordination would provide valuable insights into the systemic regulation of aging.

In conclusion, our study has uncovered a novel autophagy‐independent function of ATG‐18 in gonadal longevity, revealing the complexity of lifespan regulation and highlighting the multifaceted roles of ATG proteins beyond their canonical functions. These findings open new avenues for understanding the underlying mechanisms that determine lifespan and may ultimately help in the development of interventions to promote healthy aging.

## 4 Methods

### 4.1 *C. elegans* Growth Conditions and Strains

The nematodes were cultivated at 20°C following conventional protocols on NGM plates with *Escherichia coli* OP50 strain except when specifically indicated otherwise (Brenner 1974). Strains carrying the temperature‐sensitive *glp‐1(e2141)* mutation were kept at 15°C and transferred to 25°C to activate the germline‐deficient phenotype. For comparative analyses, N2 control animals were subjected to the same temperature shift protocol (15°C–25°C) to ensure identical experimental conditions. Mutants and transgenic strains were outcrossed with N2 (wild‐type) animals four times before use. The complete list of strains utilized in this investigation is provided in Table S1. The primers used for genotyping mutants are listed in Table S2.

### 4.2 Construction of Transgenic Strains

For *rab‐3p::atg‐18::gfp* and *ges‐1p::atg‐18::gfp* translational fusion constructs, the *rab‐3* 3 kb and *ges‐1* 3 kb endogenous promoter and *atg‐18* endogenous coding sequence were cloned into a pDC4 vector containing the EGFP tag. The transgenic line *dkIs1083[rab‐3p::atg‐18::GFP::atg‐18 utr, unc‐119(+)]; unc‐119(ed3)* were generated using the microparticle bombardment method, as previously described (Praitis et al. 2001). The transgenic line *nakEx35[ges‐1p::atg‐18::GFP, myo‐2p::mCherry]* was generated by microinjection using *myo‐2p::mCherry* as a coinjection marker.

### 4.3 RNAi Treatment

RNA interference was performed by feeding nematodes with HT115 (DE3) bacterial cells transformed with the L4440 vector, which promotes the synthesis of double‐stranded RNA targeting the gene of interest. Age‐synchronized worms were reared on RNAi plates supplemented with isopropyl β‐D‐thiogalactopyranoside and ampicillin. The RNAi bacterial strains were sourced from the Ahringer *C. elegans* RNAi feeding library. As a non‐specific control, RNAi directed against Luciferase (L4440::Luc) was employed.

### 4.4 Lifespan Analysis

Synchronized worm populations were established through 6‐h egg‐laying periods on NGM plates containing either OP50 bacteria or RNAi bacteria. When worms reached Day 1 of adulthood, 120 individuals were distributed across six plates containing 2′‐Deoxy‐5‐fluorouridine (FUdR) and maintained at 20°C. At Day 5 of adulthood, the nematodes were transferred to plates without FUdR and subsequently moved to fresh plates at regular intervals. Survival was monitored every 2–3 days. For experiments using the *glp‐1(e2141)* temperature‐sensitive mutants, synchronized eggs were incubated at 25°C for 2–3 days. Then, they were transferred to plates without FUdR and incubated at 20°C from day one of adulthood onwards. Worm viability was determined by their response to physical stimulation with a platinum wire probe, with unresponsive animals recorded as dead. Animals showing internal hatching, vulval rupture, or those that crawled off the plates were censored from the analysis. The mean, median, and maximum lifespans were assessed using the Kaplan–Meier estimation, and a log‐rank test was conducted for statistical analysis. Each experiment was replicated two to three times (Table S3).

### 4.5 Microscopy

For the quantitative analysis of ATG‐18::GFP and PCK‐2::YFP fluorescence, nematodes were immobilized using 0.1% sodium azide. Images were acquired at consistent exposure settings using an FV3000 confocal microscope (Olympus), FV4000 confocal microscope (Olympus), or SZX16 stereomicroscope (Olympus). ImageJ software (Wayne Rasband) was employed to measure and quantify fluorescence intensities in various anatomical regions: nerve‐ring neurons (ATG‐18::GFP), intestine (ATG‐18::GFP and PCK‐2::YFP), whole body (PCK‐2::YFP).

### 4.6 Autophagy Flux Assay

Day 1 adult worms expressing the GFP::LGG‐1 or CERULEAN‐VENUS::LGG‐1 fusion protein were placed on RNAi plates with or without 5 mM chloroquine for a 24‐h incubation period to impair autophagic flux by inhibiting autophagosome‐lysosome fusion in various tissues. The nematodes were immobilized using 0.1% sodium azide and positioned on a 5% agarose pad. Images were captured using either an FV3000 or FV4000 confocal microscope (Olympus). The imaging process included 10 animals per condition, and all experiments were independently repeated three times.

### 4.7 RNA Extraction and qRT‐PCR

Samples of *C. elegans* were collected in QIAZOL reagent (QIAGEN) at different developmental stages. For each experimental condition, approximately 200–300 nematodes were gathered, and RNA was isolated using the RNeasy mini kit (QIAGEN). The extracted RNA was then reverse transcribed to cDNA utilizing the iScript system (Bio‐Rad). Quantitative real‐time PCR was conducted with Power SYBR Green (Applied Biosystems) on a QuantStudio 7 Flex Real‐Time PCR System (Thermo Fisher Scientific). Each experiment included three technical replicates. The *ama‐1* gene served as the normalization control. The qRT‐PCR primer sequences are included in Table S2.

### 4.8 Western Blotting

The worms were homogenized in lysis buffer composed of 50 mM Tris/HCl (pH 7.4), 150 mM NaCl, 1 mM EDTA, and 0.1% NP‐40. Following centrifugation, the collected supernatants were heated to 95°C for 7 min. Protein samples were resolved by SDS‐PAGE and subsequently transferred to PVDF membranes, which were then blocked before incubation with specific primary antibodies. The following primary antibodies were used at the indicated dilutions: GFP (Clontech, 632375, 1/2000) and α‐Tubulin (Sigma‐Aldrich, A11126, 1:10,000). Band intensities were quantified using Image Lab software (Bio‐Rad).

### 4.9 Interactome Analysis of ATG‐18::GFP

Three experimental conditions were analyzed: (1) N2 without GFP as a negative control, (2) N2 expressing ATG‐18::GFP, and (3) *glp‐1* expressing ATG‐18::GFP. All strains were subjected to the same temperature shift protocol (15°C to 25°C). Immunoprecipitation for the interactome analysis using GFP‐Trap was performed as previously described (Yanagawa et al. 2024). Briefly, the collected supernatants described as above were incubated with GFP‐Trap magnetic agarose beads (gtma‐20; ChromoTek) at 4°C for 2 h. The beads were washed four times with the lysis buffer and then twice with 50 mM ammonium bicarbonate. Proteins on the beads were digested by adding 200 ng of trypsin (Thermo Fisher Scientific) at 37°C overnight. The resultant digests were reduced, alkylated, acidified, purified by the SP3 method, and eluted in 0.1% trifluoroacetic acid and 2% DMSO.

Liquid chromatography with tandem mass spectrometry (LC–MS/MS) analysis of the resultant peptides was performed on an EASY‐nLC 1200 UHPLC connected to an Orbitrap Fusion mass spectrometer through a nanoelectrospray ion source (Thermo Fisher Scientific). The peptides were separated on a C18 reversed‐phase column (75 μm × 150 mm; Nikkyo Technos) with a linear 4%–32% ACN gradient for 0–100 min, followed by an increase to 80% ACN for 10 min and a final hold at 80% ACN for 10 min. The mass spectrometer was operated in data‐dependent acquisition mode with a maximum duty cycle of 3 s. MS1 spectra were measured with a resolution of 120,000, an automatic gain control target of 4 × 10^5^ and a mass range of 375–1500 m/z. Higher‐energy collisional dissociation (HCD) MS/MS spectra were acquired in the linear ion trap with an automatic gain control target of 1 × 10^4^, an isolation window of 1.6 m/z, a maximum injection time of 35 ms and a normalized collision energy of 30. Dynamic exclusion was set to 20 s.

The raw data were analyzed using the MaxQuant quantitative software package, versions 2.1.3.0 (Cox and Mann 2008). Peptide and protein level identification were both set to a false discovery rate of 1% using a target‐decoy based strategy. The database supplied to the search engine for peptide identifications was the *C. elegans* Swissprot database (Release 2022_03). The mass tolerance was set to 4.5 ppm for precursor ions and MS/MS mass tolerance was set at 20 ppm. Enzyme was set to trypsin (cleavage C‐terminal to lysine and arginine) with up to two missed cleavages. Oxidation of Met and protein N‐terminal acetylation were set as variable modifications. Carbamidomethylation on Cys was searched as a fixed modification. The label‐free quantitation (LFQ) algorithm in MaxQuant was used for protein quantitation. The ‘protein groups’ file as MaxQuant output was analyzed further in RStudio with the R package of DEP for differential expression/enrichment analysis with moderated *t*‐test from limma for proteomics data (Ritchie et al. 2015; Zhang et al. 2018).

Volcano plots were generated using R (version 4.4.0) and ggplot2 (version 3.5.1) to visualize differential protein expression. ATG‐18 interactor candidates were selected based on two criteria: significant enrichment in glp‐1; ATG‐18::GFP versus N2 control (log2 fold‐change > |1|, *p*‐value < 0.05) to exclude non‐specific binding to beads, and significant enrichment in glp‐1; ATG‐18::GFP versus N2; ATG‐18::GFP (log2 fold‐change > 1, *p*‐value < 0.05) to identify interactors specifically enriched upon germline deficiency.

### 4.10 Survival Screening

To identify ATG‐18 interactors relevant to gonadal longevity, we performed survival screening using *glp‐1; rrf‐3* double mutants with enhanced RNAi sensitivity. Candidate genes identified from proteomics analysis were subjected to RNAi knockdown. Synchronized animals were cultured on RNAi plates from hatching, and survival was assessed at day 25 of adulthood. Approximately 50 animals were examined per condition. Relative survival rate was calculated by normalizing to the survival rate of *glp‐1; rrf‐3* animals fed with control RNAi (L4440::Luc). Candidates showing relative survival rate < 0.6 were considered as potential regulators of gonadal longevity and subjected to further validation by full lifespan analysis.

### 4.11 Co‐IP Experiment

HEK293 cells were cultured in Dulbecco's Modified Eagle's Medium (DMEM; high glucose) supplemented with 10% fetal bovine serum (FBS) and penicillin–streptomycin at 37°C in a humidified atmosphere containing 5% CO_2_. Plasmids were transfected into cells using Lipofectamine 2000 (Invitrogen) according to the manufacturer's instructions. Cells were harvested 48 h after transfection. Plasmids used in this study (Table S4) were generated by gene synthesis (VectorBuilder). The coding sequence of *C. elegans* ATG‐18 and PCK‐2 was codon‐optimized for expression in human cells. Prior to lysis, transfected cells were washed twice with ice‐cold PBS and lysed in M‐PER Mammalian Protein Extraction Reagent (Thermo Scientific) supplemented with 1× Complete protease inhibitor cocktail (Roche) and 1 mM phenylmethylsulfonyl fluoride (PMSF; Sigma‐Aldrich). Lysates were clarified by centrifugation at 15,000 × g for 10 min at 4°C. The supernatants were incubated with anti‐FLAG M2 magnetic beads (Millipore) at 4°C for 2 h. Beads were washed three times with TBS, eluted with SDS sample buffer, and subjected to immunoblot analysis. Proteins were separated by SDS–PAGE and transferred onto PVDF membranes. Membranes were blocked with TBST containing 1% skim milk and incubated overnight at 4°C with primary antibodies diluted in TBST containing 1% skim milk: anti‐FLAG (mouse monoclonal, 1:1000; Sigma‐Aldrich, F1804) and anti‐HA (rat monoclonal, 1:1000; Roche, 11867423001). Membranes were washed three times with TBST, incubated for 1 h at room temperature with HRP‐conjugated secondary antibodies (1:5000 diluted in TBST containing 1% skim milk), washed three times with TBST, and immunoreactive bands were visualized using a ChemiDoc Touch imaging system (Bio‐Rad).

### 4.12 Statistical Analysis

The experimental results were performed using GraphPad Prism 10 software (GraphPad Software) or Microsoft Excel (Microsoft). Lifespan curves and graphs were generated using GraphPad Prism software. Statistical analysis was performed using either the *t*‐test or one‐way ANOVA with Tukey's test. Data are presented as vertical scatter plots with mean ± SD.

## Author Contributions

T. Shioda and S.N. designed research; T. Shioda, I.T., M.H., A.Y., T.O., T. Shima, K.N., T.K., H.Y., and H.K. performed research; M.H., T. Sasaki, M.S., and H.K. contributed new reagents/analytic tools; T. Shioda, I.T., and H.Y. analyzed data; T. Shioda, T.Y., and S.N. funding acquisition; T.Y. and S.N. project administration; and T. Shioda, I.T., and S.N. wrote the paper.

## Funding

T.Y. is supported by Japan Society for the Promotion of Science (JSPS) KAKENHI (22H04982), Japan Agency for Medical Research and Development (AMED) (grant nos. JP22gm1410014, JP25gm0010012), and the Takeda Science Foundation. S.N. is supported by AMED (JP24gm1910008, JP25gm0010012), JSPS KAKENHI (24H01910, 24K01979), The Uehara Memorial Foundation, Chugai Foundation for Innovative Drug Discovery Science, The Naito Foundation, Toray Science Foundation (23‐6408), the Cell Science Research Foundation, Takeda Science Foundation, Joint Usage and Joint Research Programs of the Institute of Advanced Medical Sciences of Tokushima University, and Center for Autophagy and Anti‐Aging Research, Nara Medical University. M.H. is supported by Visionary Research Grants (Start) from Takeda Science Foundation. T.S. is supported by JSPS KAKENHI (22J13562). This work was also supported by Medical Research Center Initiative for High Depth Omics.

## Conflicts of Interest

T.Y. and S.N. are founders of AutoPhagyGO.

## Supporting information


**Table S1:** *C. elegans* strains.

**Table S2:** Primers.

**Table S3:** Lifespan analysis.

**Table S4:** Plasmid.

**Figure S1–S8:** acel70454‐sup‐0005‐FigureS1‐S8.

## Data Availability

The MS proteomics data have been deposited to the ProteomeXchange Consortium via the jPOST partner repository with the dataset identifier PXD066526 (Okuda et al. 2025).

## References

[acel70454-bib-0001] Arantes‐Oliveira, N. , J. Apfeld , A. Dillin , and C. Kenyon . 2002. “Regulation of Life‐Span by Germ‐Line Stem Cells in *Caenorhabditis elegans* .” Science 295, no. 5554: 502–505. 11799246 10.1126/science.1065768

[acel70454-bib-0002] Baskaran, S. , M. J. Ragusa , E. Boura , and J. H. Hurley . 2012. “Two‐Site Recognition of Phosphatidylinositol 3‐Phosphate by PROPPINs in Autophagy.” Molecular Cell 47, no. 3: 339–348. 22704557 10.1016/j.molcel.2012.05.027 PMC3595537

[acel70454-bib-0003] Brenner, S. 1974. “The Genetics of *Caenorhabditis elegans* .” Genetics 77, no. 1: 71–94. 4366476 10.1093/genetics/77.1.71 PMC1213120

[acel70454-bib-0004] Burgess, S. C. , N. Hausler , M. Merritt , et al. 2004. “Impaired Tricarboxylic Acid Cycle Activity in Mouse Livers Lacking Cytosolic Phosphoenolpyruvate Carboxykinase.” Journal of Biological Chemistry 279, no. 47: 48941–48949. 15347677 10.1074/jbc.M407120200

[acel70454-bib-0005] Calixto, A. , D. Chelur , I. Topalidou , X. Chen , and M. Chalfie . 2010. “Enhanced Neuronal RNAi in *C. elegans* Using SID‐1.” Nature Methods 7, no. 7: 554–559. 20512143 10.1038/nmeth.1463 PMC2894993

[acel70454-bib-0006] Chang, J. T. , C. Kumsta , A. B. Hellman , L. M. Adams , and M. Hansen . 2017. “Spatiotemporal Regulation of Autophagy During *Caenorhabditis elegans* Aging.” eLife 6: 6. 10.7554/eLife.18459 PMC5496740 28675140

[acel70454-bib-0007] Chaturbedi, A. , and S. S. Lee . 2025. “Different Gametogenesis States Uniquely Impact Longevity in *Caenorhabditis elegans* .” Nature Communications 16: 9300. 10.1038/s41467-025-64341-x PMC12541034 41120259

[acel70454-bib-0008] Chen, Y. L. , J. Tao , P. J. Zhao , et al. 2019. “Adiponectin Receptor PAQR‐2 Signaling Senses Low Temperature to Promote *C. elegans* Longevity by Regulating Autophagy.” Nature Communications 10, no. 1: 2602. 10.1038/s41467-019-10475-8 PMC6565724 31197136

[acel70454-bib-0009] Courtellemont, T. , M. G. de Leo , N. Gopaldass , and A. Mayer . 2022. “CROP: A Retromer‐PROPPIN Complex Mediating Membrane Fission in the Endo‐Lysosomal System.” EMBO Journal 41, no. 10: e109646. 35466426 10.15252/embj.2021109646 PMC9108610

[acel70454-bib-0010] Cox, J. , and M. Mann . 2008. “MaxQuant Enables High Peptide Identification Rates, Individualized p.p.b.‐Range Mass Accuracies and Proteome‐Wide Protein Quantification.” Nature Biotechnology 26, no. 12: 1367–1372. 10.1038/nbt.1511 19029910

[acel70454-bib-0011] Dahlberg, C. L. , and P. Juo . 2014. “The WD40‐Repeat Proteins WDR‐20 and WDR‐48 Bind and Activate the Deubiquitinating Enzyme USP‐46 to Promote the Abundance of the Glutamate Receptor GLR‐1 in the Ventral Nerve Cord of *Caenorhabditis elegans* .” Journal of Biological Chemistry 289, no. 6: 3444–3456. 24356955 10.1074/jbc.M113.507541 PMC3916546

[acel70454-bib-0012] Eisenberg, T. , H. Knauer , A. Schauer , et al. 2009. “Induction of Autophagy by Spermidine Promotes Longevity.” Nature Cell Biology 11, no. 11: 1305–1314. 19801973 10.1038/ncb1975

[acel70454-bib-0013] Espelt, M. V. , A. Y. Estevez , X. Yin , and K. Strange . 2005. “Oscillatory Ca2+ Signaling in the Isolated *Caenorhabditis elegans* Intestine: Role of the Inositol‐1,4,5‐Trisphosphate Receptor and Phospholipases C Beta and Gamma.” Journal of General Physiology 126, no. 4: 379–392. 16186564 10.1085/jgp.200509355 PMC2266627

[acel70454-bib-0072] Fernández, Á. F. , S. Sebti , Y. Wei , et al. 2018. “Disruption of the Beclin 1–BCL2 Autophagy Regulatory Complex Promotes Longevity in Mice.” Nature 558, no. 7708: 136–140. 10.1038/s41586-018-0162-7. 29849149 PMC5992097

[acel70454-bib-0014] Franckhauser, S. , S. Muñoz , I. Elias , T. Ferre , and F. Bosch . 2006. “Adipose Overexpression of Phosphoenolpyruvate Carboxykinase Leads to High Susceptibility to Diet‐Induced Insulin Resistance and Obesity.” Diabetes 55, no. 2: 273–280. 16443757 10.2337/diabetes.55.02.06.db05-0482

[acel70454-bib-0015] Franckhauser, S. , S. Muñoz , A. Pujol , et al. 2002. “Increased Fatty Acid Re‐Esterification by PEPCK Overexpression in Adipose Tissue Leads to Obesity Without Insulin Resistance.” Diabetes 51, no. 3: 624–630. 11872659 10.2337/diabetes.51.3.624

[acel70454-bib-0016] Franco‐Romero, A. , V. Morbidoni , G. Milan , et al. 2024. “C16ORF70/MYTHO Promotes Healthy Aging in *C. elegans* and Prevents Cellular Senescence in Mammals.” Journal of Clinical Investigation 134, no. 15: e165814. 38869949 10.1172/JCI165814 PMC11291266

[acel70454-bib-0017] Gahlot, S. , and J. Singh . 2024. “ *Caenorhabditis elegans* Neuronal RNAi Does Not Render Other Tissues Refractory to RNAi.” Proceedings of the National Academy of Sciences of the United States of America 121, no. 22: e2401096121. 38768358 10.1073/pnas.2401096121 PMC11145236

[acel70454-bib-0018] Galluzzi, L. , and D. R. Green . 2019. “Autophagy‐Independent Functions of the Autophagy Machinery.” Cell 177, no. 7: 1682–1699. 31199916 10.1016/j.cell.2019.05.026 PMC7173070

[acel70454-bib-0019] Gelino, S. , J. T. Chang , C. Kumsta , et al. 2016. “Intestinal Autophagy Improves Healthspan and Longevity in *C. elegans* During Dietary Restriction.” PLoS Genetics 12, no. 7: e1006135. 27414651 10.1371/journal.pgen.1006135 PMC4945006

[acel70454-bib-0020] Hansen, M. , T. Flatt , and H. Aguilaniu . 2013. “Reproduction, Fat Metabolism, and Life Span: What Is the Connection?” Cell Metabolism 17, no. 1: 10–19. 23312280 10.1016/j.cmet.2012.12.003 PMC3567776

[acel70454-bib-0021] Harrison, D. E. , R. Strong , Z. D. Sharp , et al. 2009. “Rapamycin Fed Late in Life Extends Lifespan in Genetically Heterogeneous Mice.” Nature 460, no. 7253: 392–395. 19587680 10.1038/nature08221 PMC2786175

[acel70454-bib-0022] Held, J. P. , N. H. Dbouk , A. M. Strozak , et al. 2024. “Germline Status and Micronutrient Availability Regulate a Somatic Mitochondrial Quality Control Pathway via Short‐Chain Fatty Acid Metabolism.” *bioRxiv*. 10.1101/2024.05.20.594820.

[acel70454-bib-0023] Hsin, H. , and C. Kenyon . 1999. “Signals From the Reproductive System Regulate the Lifespan of *C. elegans* .” Nature 399, no. 6734: 362–366. 10360574 10.1038/20694

[acel70454-bib-0024] Hu, Z. , Y. Luo , Y. Liu , et al. 2023. “Partial Inhibition of Class III PI3K VPS‐34 Ameliorates Motor Aging and Prolongs Health Span.” PLoS Biology 21, no. 7: e3002165. 37432924 10.1371/journal.pbio.3002165 PMC10335676

[acel70454-bib-0025] Jia, K. , and B. Levine . 2007. “Autophagy Is Required for Dietary Restriction‐Mediated Life Span Extension in *C. elegans* .” Autophagy 3, no. 6: 597–599. 17912023 10.4161/auto.4989

[acel70454-bib-0026] Jia, R. , J. Zhang , and K. Jia . 2019. “Neuroendocrine Regulation of Fat Metabolism by Autophagy Gene Atg‐18 in *C. elegans* Dauer Larvae.” FEBS Open Bio 9, no. 9: 1623–1631. 10.1002/2211-5463.12708 PMC6722879 31368651

[acel70454-bib-0027] Joo, J. H. , B. Wang , E. Frankel , et al. 2016. “The Noncanonical Role of ULK/ATG1 in ER‐To‐Golgi Trafficking Is Essential for Cellular Homeostasis.” Molecular Cell 62, no. 4: 491–506. 27203176 10.1016/j.molcel.2016.04.020 PMC4993601

[acel70454-bib-0028] Jung, R. , M. C. Lechler , A. Fernandez‐Villegas , et al. 2023. “A Safety Mechanism Enables Tissue‐Specific Resistance to Protein Aggregation During Aging in *C. elegans* .” PLoS Biology 21, no. 9: e3002284. 37708127 10.1371/journal.pbio.3002284 PMC10501630

[acel70454-bib-0029] Khodakarami, A. , I. Saez , J. Mels , and D. Vilchez . 2015. “Mediation of Organismal Aging and Somatic Proteostasis by the Germline.” Frontiers in Molecular Biosciences 2: 3. 25988171 10.3389/fmolb.2015.00003 PMC4428440

[acel70454-bib-0030] Klionsky, D. J. , A. K. Abdel‐Aziz , S. Abdelfatah , et al. 2021. “Guidelines for the Use and Interpretation of Assays for Monitoring Autophagy (4th Edition) (1).” Autophagy 17, no. 1: 1–382. 33634751 10.1080/15548627.2020.1797280 PMC7996087

[acel70454-bib-0031] Kuma, A. , M. Hatano , M. Matsui , et al. 2004. “The Role of Autophagy During the Early Neonatal Starvation Period.” Nature 432, no. 7020: 1032–1036. 15525940 10.1038/nature03029

[acel70454-bib-0032] Kumsta, C. , J. T. Chang , R. Lee , et al. 2019. “The Autophagy Receptor p62/SQST‐1 Promotes Proteostasis and Longevity in *C. elegans* by Inducing Autophagy.” Nature Communications 10, no. 1: 5648. 10.1038/s41467-019-13540-4 PMC6906454 31827090

[acel70454-bib-0033] Lapierre, L. R. , C. D. de Magalhaes Filho , P. R. McQuary , et al. 2013. “The TFEB Orthologue HLH‐30 Regulates Autophagy and Modulates Longevity in *Caenorhabditis elegans* .” Nature Communications 4: 2267. 10.1038/ncomms3267 PMC3866206 23925298

[acel70454-bib-0034] Lapierre, L. R. , S. Gelino , A. Meléndez , and M. Hansen . 2011. “Autophagy and Lipid Metabolism Coordinately Modulate Life Span in Germline‐Less *C. elegans* .” Current Biology 21, no. 18: 1507–1514. 21906946 10.1016/j.cub.2011.07.042 PMC3191188

[acel70454-bib-0035] Lee, I. H. , Y. Kawai , M. M. Fergusson , et al. 2012. “Atg7 Modulates p53 Activity to Regulate Cell Cycle and Survival During Metabolic Stress.” Science 336, no. 6078: 225–228. 22499945 10.1126/science.1218395 PMC4721513

[acel70454-bib-0036] Lu, Q. , P. Yang , X. Huang , et al. 2011. “The WD40 Repeat PtdIns(3)P‐Binding Protein EPG‐6 Regulates Progression of Omegasomes to Autophagosomes.” Developmental Cell 21, no. 2: 343–357. 21802374 10.1016/j.devcel.2011.06.024

[acel70454-bib-0037] Meléndez, A. , Z. Tallóczy , M. Seaman , E. L. Eskelinen , D. H. Hall , and B. Levine . 2003. “Autophagy Genes Are Essential for Dauer Development and Life‐Span Extension in *C. elegans* .” Science 301, no. 5638: 1387–1391. 12958363 10.1126/science.1087782

[acel70454-bib-0038] Metcalf, M. G. , S. Monshietehadi , A. Sahay , et al. 2024. “Cell Non‐Autonomous Control of Autophagy and Metabolism by Glial Cells.” iScience 27, no. 4: 109354. 38500817 10.1016/j.isci.2024.109354 PMC10946330

[acel70454-bib-0039] Minnerly, J. , J. Zhang , T. Parker , T. Kaul , and K. Jia . 2017. “The Cell Non‐Autonomous Function of ATG‐18 Is Essential for Neuroendocrine Regulation of *Caenorhabditis elegans* Lifespan.” PLoS Genetics 13, no. 5: e1006764. 28557996 10.1371/journal.pgen.1006764 PMC5469504

[acel70454-bib-0040] Mizushima, N. , T. Yoshimori , and Y. Ohsumi . 2011. “The Role of Atg Proteins in Autophagosome Formation.” Annual Review of Cell and Developmental Biology 27: 107–132. 10.1146/annurev-cellbio-092910-154005 21801009

[acel70454-bib-0041] Nakamura, S. , M. Oba , M. Suzuki , et al. 2019. “Suppression of Autophagic Activity by Rubicon Is a Signature of Aging.” Nature Communications 10, no. 1: 847. 10.1038/s41467-019-08729-6 PMC6381146 30783089

[acel70454-bib-0042] Nakamura, S. , and T. Yoshimori . 2018. “Autophagy and Longevity.” Molecules and Cells 41, no. 1: 65–72. 29370695 10.14348/molcells.2018.2333 PMC5792715

[acel70454-bib-0043] Nishimura, T. , and S. A. Tooze . 2020. “Emerging Roles of ATG Proteins and Membrane Lipids in Autophagosome Formation.” Cell Discovery 6, no. 1: 32. 32509328 10.1038/s41421-020-0161-3 PMC7248066

[acel70454-bib-0044] Obara, K. , T. Sekito , K. Niimi , and Y. Ohsumi . 2008. “The Atg18‐Atg2 Complex Is Recruited to Autophagic Membranes via Phosphatidylinositol 3‐Phosphate and Exerts an Essential Function.” Journal of Biological Chemistry 283, no. 35: 23972–23980. 18586673 10.1074/jbc.M803180200 PMC3259791

[acel70454-bib-0045] Okuda, S. , A. C. Yoshizawa , D. Kobayashi , et al. 2025. “jPOST Environment Accelerates the Reuse and Reanalysis of Public Proteome Mass Spectrometry Data.” Nucleic Acids Research 53, no. D1: D462–d467. 39526391 10.1093/nar/gkae1032 PMC11701591

[acel70454-bib-0046] Olsen, A. , and M. S. Gill . 2017. Ageing: Lessons From C. elegans. Vol. 5. Springer.

[acel70454-bib-0047] Olswang, Y. , H. Cohen , O. Papo , et al. 2002. “A Mutation in the Peroxisome Proliferator‐Activated Receptor Gamma‐Binding Site in the Gene for the Cytosolic Form of Phosphoenolpyruvate Carboxykinase Reduces Adipose Tissue Size and Fat Content in Mice.” Proceedings of the National Academy of Sciences of the United States of America 99, no. 2: 625–630. 11792850 10.1073/pnas.022616299 PMC117356

[acel70454-bib-0048] Onken, B. , N. Kalinava , and M. Driscoll . 2020. “Gluconeogenesis and PEPCK Are Critical Components of Healthy Aging and Dietary Restriction Life Extension.” PLoS Genetics 16, no. 8: e1008982. 32841230 10.1371/journal.pgen.1008982 PMC7473531

[acel70454-bib-0049] O'Rourke, E. J. , A. A. Soukas , C. E. Carr , and G. Ruvkun . 2009. “ *C. elegans* Major Fats Are Stored in Vesicles Distinct From Lysosome‐Related Organelles.” Cell Metabolism 10, no. 5: 430–435. 19883620 10.1016/j.cmet.2009.10.002 PMC2921818

[acel70454-bib-0050] Possik, E. , L. L. Klein , P. Sanjab , et al. 2023. “Glycerol 3‐Phosphate Phosphatase/PGPH‐2 Counters Metabolic Stress and Promotes Healthy Aging via a Glycogen Sensing‐AMPK‐HLH‐30‐Autophagy Axis in *C. elegans* .” Nature Communications 14, no. 1: 5214. 10.1038/s41467-023-40857-y PMC10457390 37626039

[acel70454-bib-0051] Praitis, V. , E. Casey , D. Collar , and J. Austin . 2001. “Creation of Low‐Copy Integrated Transgenic Lines in *Caenorhabditis elegans* .” Genetics 157, no. 3: 1217–1226. 11238406 10.1093/genetics/157.3.1217 PMC1461581

[acel70454-bib-0052] Pyo, J. O. , S. M. Yoo , H. H. Ahn , et al. 2013. “Overexpression of Atg5 in Mice Activates Autophagy and Extends Lifespan.” Nature Communications 4: 2300. 10.1038/ncomms3300 PMC3753544 23939249

[acel70454-bib-0053] Qadota, H. , M. Inoue , T. Hikita , et al. 2007. “Establishment of a Tissue‐Specific RNAi System in *C. elegans* .” Gene 400, no. 1–2: 166–173. 17681718 10.1016/j.gene.2007.06.020 PMC3086655

[acel70454-bib-0054] Ritchie, M. E. , B. Phipson , D. Wu , et al. 2015. “Limma Powers Differential Expression Analyses for RNA‐Sequencing and Microarray Studies.” Nucleic Acids Research 43, no. 7: e47. 25605792 10.1093/nar/gkv007 PMC4402510

[acel70454-bib-0055] Ryu, D. , L. Mouchiroud , P. A. Andreux , et al. 2016. “Urolithin A Induces Mitophagy and Prolongs Lifespan in *C. elegans* and Increases Muscle Function in Rodents.” Nature Medicine 22, no. 8: 879–888. 10.1038/nm.4132 27400265

[acel70454-bib-0056] Shang, J. N. , C. G. Yu , R. Li , et al. 2024. “The Nonautophagic Functions of Autophagy‐Related Proteins.” Autophagy 20, no. 4: 720–734. 37682088 10.1080/15548627.2023.2254664 PMC11062363

[acel70454-bib-0057] Shioda, T. , I. Takahashi , K. Ikenaka , et al. 2023. “Neuronal MML‐1/MXL‐2 Regulates Systemic Aging via Glutamate Transporter and Cell Nonautonomous Autophagic and Peroxidase Activity.” Proceedings of the National Academy of Sciences of the United States of America 120, no. 39: e2221553120. 37722055 10.1073/pnas.2221553120 PMC10523562

[acel70454-bib-0073] Simmer, F. , C. Tijsterman , S. Parrish , et al. 2002. “Loss of the Putative RNA‐Directed RNA Polymerase RRF‐3 Makes *C. elegans* Hypersensitive to RNAi.” Current Biology 12, no. 15: 1317–1319. 12176360 10.1016/s0960-9822(02)01041-2

[acel70454-bib-0058] Singh, A. , B. Kaduskar , K. C. Reddy , et al. 2022. “Conserved Neuropeptidergic Regulation of Intestinal Integrity in Invertebrate Models of Aging.” *bioRxiv*. 10.1101/2022.02.24.481867.

[acel70454-bib-0059] Subramani, S. , and V. Malhotra . 2013. “Non‐Autophagic Roles of Autophagy‐Related Proteins.” EMBO Reports 14, no. 2: 143–151. 23337627 10.1038/embor.2012.220 PMC3566844

[acel70454-bib-0060] Takacs, Z. , K. Sporbeck , J. Stoeckle , M. J. Prado Carvajal , M. Grimmel , and T. Proikas‐Cezanne . 2019. “ATG‐18 and EPG‐6 Are Both Required for Autophagy but Differentially Contribute to Lifespan Control in *Caenorhabditis elegans* .” Cells 8, no. 3: 236. 30871075 10.3390/cells8030236 PMC6468378

[acel70454-bib-0061] Tamura, N. , M. Oku , M. Ito , N. N. Noda , F. Inagaki , and Y. Sakai . 2013. “Atg18 Phosphoregulation Controls Organellar Dynamics by Modulating Its Phosphoinositide‐Binding Activity.” Journal of Cell Biology 202, no. 4: 685–698. 23940117 10.1083/jcb.201302067 PMC3747300

[acel70454-bib-0062] Tóth, M. L. , T. Sigmond , E. Borsos , et al. 2008. “Longevity Pathways Converge on Autophagy Genes to Regulate Life Span in *Caenorhabditis elegans* .” Autophagy 4, no. 3: 330–338. 18219227 10.4161/auto.5618

[acel70454-bib-0063] Tran, S. , J. Juliani , T. J. Harris , et al. 2024. “BECLIN1 Is Essential for Intestinal Homeostasis Involving Autophagy‐Independent Mechanisms Through Its Function in Endocytic Trafficking.” Communications Biology 7, no. 1: 209. 38378743 10.1038/s42003-024-05890-7 PMC10879175

[acel70454-bib-0064] Watanabe, Y. , T. Kobayashi , H. Yamamoto , et al. 2012. “Structure‐Based Analyses Reveal Distinct Binding Sites for Atg2 and Phosphoinositides in Atg18.” Journal of Biological Chemistry 287, no. 38: 31681–31690. 22851171 10.1074/jbc.M112.397570 PMC3442503

[acel70454-bib-0065] Wei, Y. , and C. Kenyon . 2016. “Roles for ROS and Hydrogen Sulfide in the Longevity Response to Germline Loss in *Caenorhabditis elegans* .” Proceedings of the National Academy of Sciences of the United States of America 113, no. 20: E2832–E2841. 27140632 10.1073/pnas.1524727113 PMC4878494

[acel70454-bib-0066] Yamawaki, T. M. , J. R. Berman , M. Suchanek‐Kavipurapu , et al. 2010. “The Somatic Reproductive Tissues of *C. elegans* Promote Longevity Through Steroid Hormone Signaling.” PLoS Biology 8, no. 8: e1000468. 20824162 10.1371/journal.pbio.1000468 PMC2930862

[acel70454-bib-0067] Yanagawa, K. , A. Kuma , M. Hamasaki , et al. 2024. “The Rubicon‐WIPI Axis Regulates Exosome Biogenesis During Ageing.” Nature Cell Biology 26, no. 9: 1558–1570. 39174742 10.1038/s41556-024-01481-0

[acel70454-bib-0068] Yang, Y. , M. L. Arnold , C. M. Lange , et al. 2024. “Autophagy Protein ATG‐16.2 and Its WD40 Domain Mediate the Beneficial Effects of Inhibiting Early‐Acting Autophagy Genes in *C. elegans* Neurons.” Nature Aging 4, no. 2: 198–212. 38177330 10.1038/s43587-023-00548-1 PMC11022750

[acel70454-bib-0069] Zhang, X. , A. H. Smits , G. B. A. van Tilburg , H. Ovaa , W. Huber , and M. Vermeulen . 2018. “Proteome‐Wide Identification of Ubiquitin Interactions Using UbIA‐MS.” Nature Protocols 13, no. 3: 530–550. 29446774 10.1038/nprot.2017.147

[acel70454-bib-0070] Zhao, Z. , B. Fux , M. Goodwin , et al. 2008. “Autophagosome‐Independent Essential Function for the Autophagy Protein Atg5 in Cellular Immunity to Intracellular Pathogens.” Cell Host & Microbe 4, no. 5: 458–469. 18996346 10.1016/j.chom.2008.10.003 PMC2682425

[acel70454-bib-0071] Zhu, Y. , M. Fujimaki , L. Snape , A. Lopez , A. Fleming , and D. C. Rubinsztein . 2024. “Loss of WIPI4 in Neurodegeneration Causes Autophagy‐Independent Ferroptosis.” Nature Cell Biology 26, no. 4: 542–551. 38454050 10.1038/s41556-024-01373-3 PMC11021183

