# Supplementary material for: Autophagy‐Independent Function of ATG‐18 Is Essential for Gonadal Longevity in Caenorhabditis elegans
Source: Aging Cell. 2026 Mar 29;25(4):e70454. doi: 10.1111/acel.70454 (PMC13092510; doi:10.1111/acel.70454)
Supplement: Supplementary file 5 — Figure S1–S8: acel70454‐sup‐0005‐FigureS1‐S8. [file ACEL-25-e70454-s004.pdf]

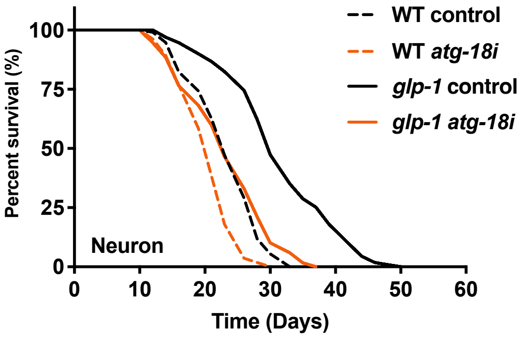

Figure S1. Neuron-specific *atg-18i* abolished *glp-1* longevity.

Lifespan analysis of wild-type (WT) and *glp-1(e2141)* animals capable of neuron-specific RNAi (MAH677) fed bacteria expressing *luciferase* (control) or *atg-18* dsRNA from adult day 1. Three biological replicates were performed with 120 worms tested per condition.

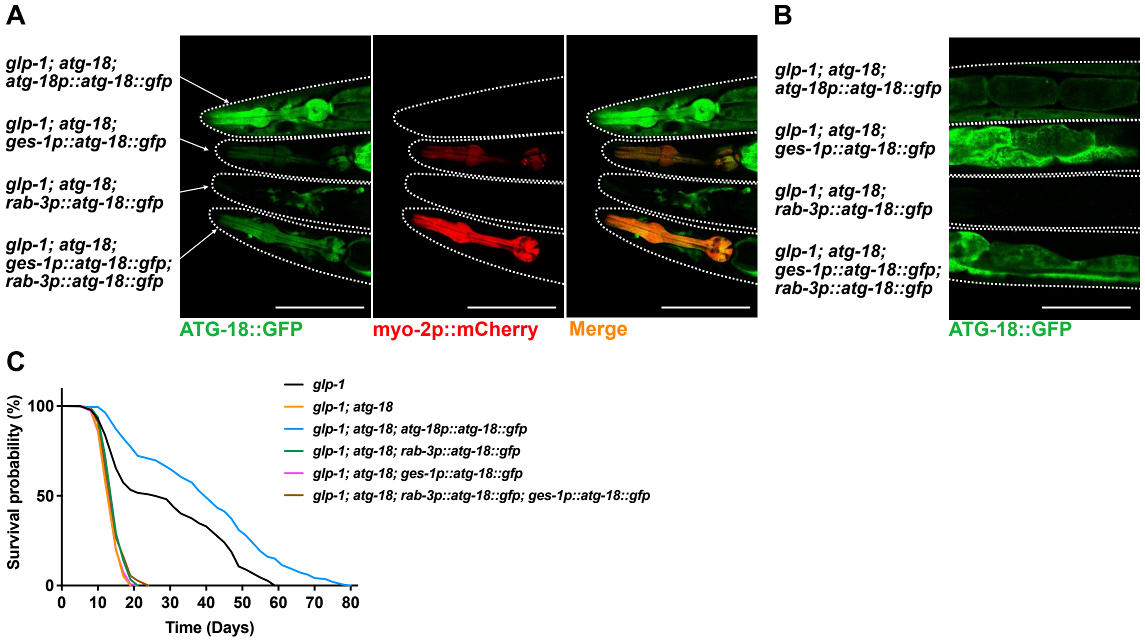

Figure S2. Tissue-specific rescue of ATG-18 does not restore the reduced lifespan of *glp-1* mutants caused by *atg-18* deletion.

(A) Representative images of ATG-18::GFP expression in the head region (nerve-ring) of *glp-1(e2141); atg-18(gk378)* mutants carrying rescue constructs driven by endogenous (*atg-18p*), intestinal (*ges-1p*), neuronal (*rab-3p*), or both intestinal and neuronal promoters. myo-2p::mCherry marks the pharynx as a co-injection marker. Dotted lines outline the body of the animal. Scale bar, 100 µm. (B) Representative images of ATG-18::GFP expression in the intestine of *glp-1(e2141); atg-18(gk378)* mutants carrying rescue constructs driven by endogenous (*atg-18p*), intestinal (*ges-1p*), neuronal (*rab-3p*), or both intestinal and neuronal promoters. Dotted lines outline the body of the animal. Scale bar, 100 µm. (C) Lifespan analysis of *glp-1(e2141), glp-1(e2141); atg-18(gk378), glp-1(e2141); atg-18(gk378); sqIs25[atg-18p::atg-18::GFP + rol-6(su1006)], glp-1(e2141); atg-18(gk378); dkIs1083[rab-3p::atg-18::GFP + unc-119(+)], glp-1(e2141); atg-18(gk378); nakEx35[ges-1p::atg-18::GFP + myo-2p::mCherry], and glp-1(e2141); atg-18(gk378); dkIs1083[rab-3p::atg-18::GFP + unc-119(+)]; nakEx35[ges-1p::atg-18::GFP + myo-2p::mCherry]* animals fed OP50 bacteria. Three biological replicates were performed with 120 worms tested per condition.

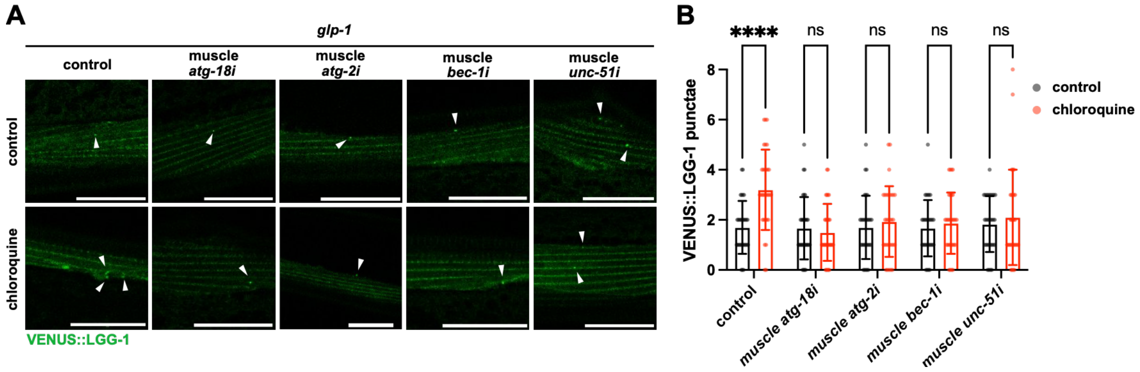

Figure S3. Muscle-specific knockdown of autophagy genes impairs autophagy flux in muscle.

(A) Representative confocal images of VENUS::LGG-1 puncta in body wall muscle of *glp-1(e2141)* mutants with muscle-specific RNAi, treated with or without 5 mM chloroquine on adult day 1. Knockdown was conducted from egg onward. Each arrow indicates VENUS::LGG-1 puncta. Scale bar, 20 μm. (B) Quantification of VENUS::LGG-1 puncta in body wall muscle shown in (A). Values represent mean ± SD (n = 30). *P* values (*ns* > 0.05, *****P* < 0.0001) were determined by two-way ANOVA with Tukey's test.

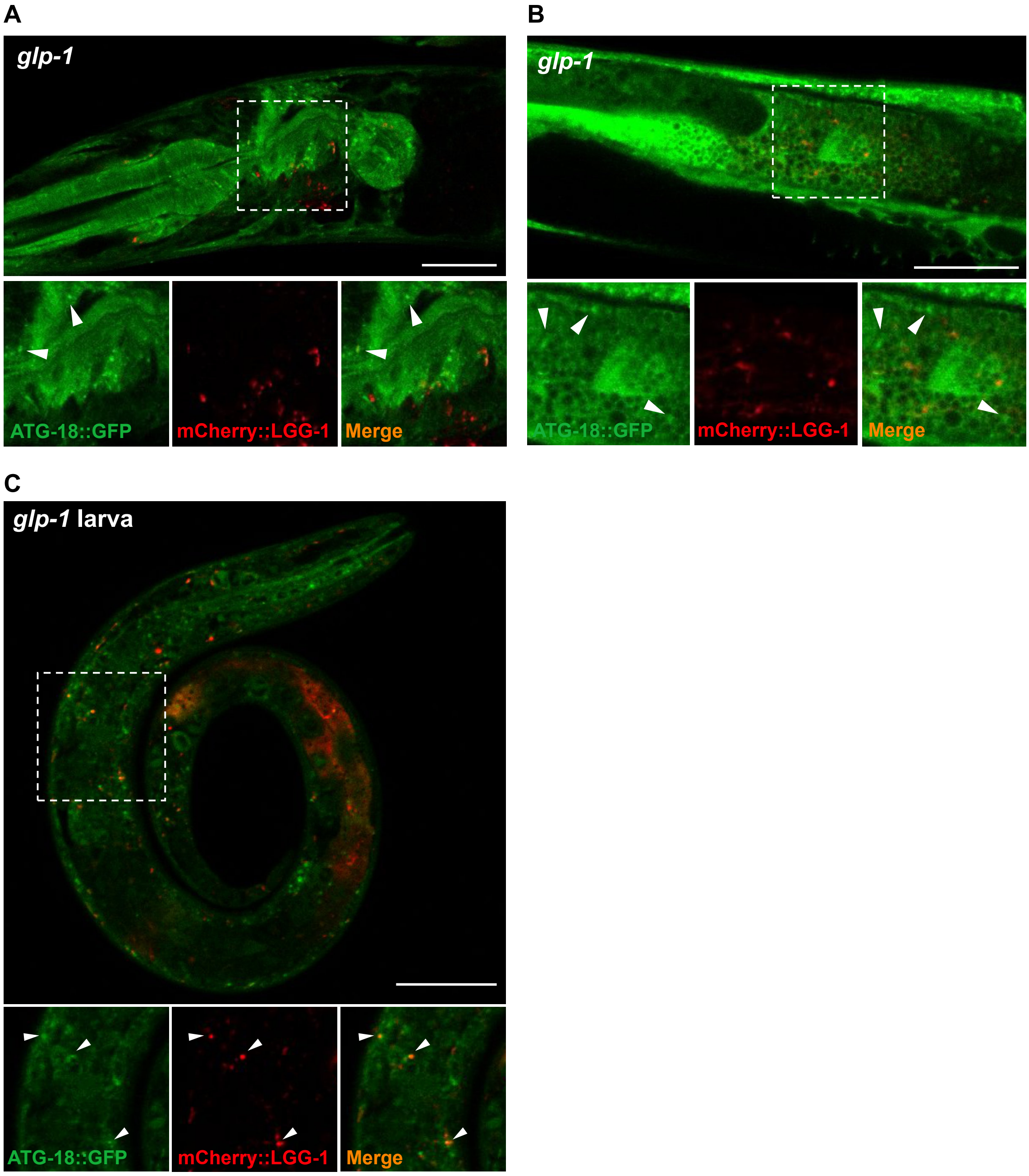

Figure S4. ATG-18 shows minimal colocalization with autophagosomes in germline deficient animals.

(A) Representative fluorescent images of ATG-18::GFP and mCherry::LGG-1 in nerve-ring neurons of *glp-1(e2141)* animals on adult day 1. Scale bars, 20 μm. (B) Representative fluorescent images of ATG-18::GFP and mCherry::LGG-1 in intestinal cells of *glp-1(e2141)* animals on adult day 1. Scale bars, 20 μm. (C) Representative fluorescent images of ATG-18::GFP and mCherry::LGG-1 during larval stages, demonstrating functional colocalization of the ATG-18::GFP reporter with autophagosomes. Scale bars, 20 μm.

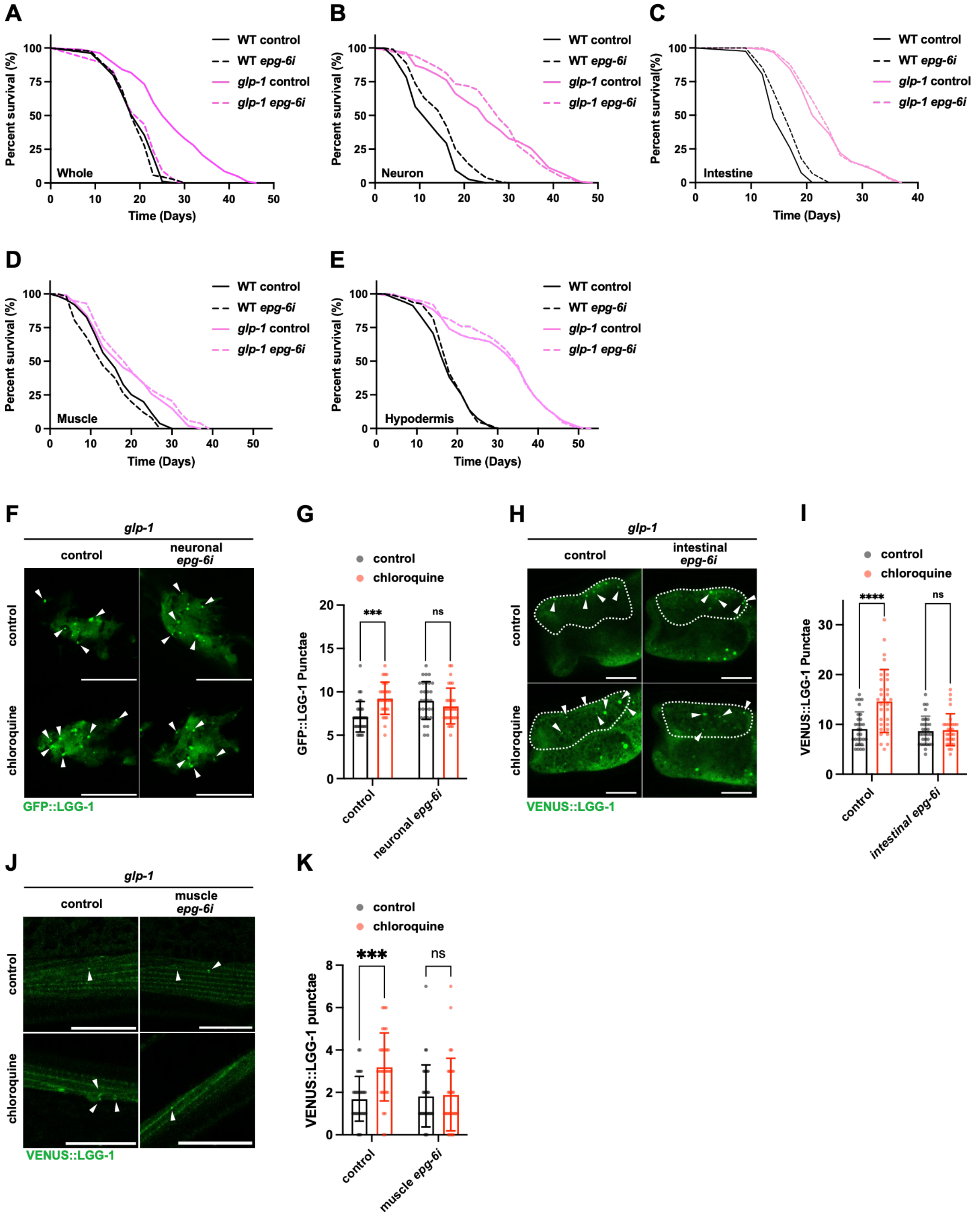

Figure S5. ***Epg-6*** **is not associated with the autophagy-independent function of** ***atg-18*** **in gonadal longevity.**

(A) Lifespan analysis of wild-type (WT) and *glp-1(e2141)* animals fed bacteria expressing *luciferase* (control) or *epg-6* dsRNA from adult day 1. Three biological replicates were performed with 120 worms tested per condition. (B-E) Lifespan analysis of wild-type (WT)

and *glp-1(e2141)* animals capable of tissue-specific RNAi (B: neuronal, C: intestinal, D: muscle, E: hypodermal) fed bacteria expressing *luciferase* (control) or *epg-6* dsRNA from adult day 1. Two biological replicates were performed with 120 worms tested per condition. (F) Representative fluorescent images of GFP::LGG-1 puncta in nerve-ring neurons of *glp-1(e2141)* animals treated with or without 5 mM chloroquine on adult day 1. Knockdown was conducted from egg onward. (G) Quantification of GFP::LGG-1 puncta in neurons shown in (F). Values represent mean ± SD (n = 30). *P* values (*ns* > 0.05, *****P* < 0.0001) were determined by two-way ANOVA with Tukey's test. (H) Representative fluorescent images of VENUS::LGG-1 puncta in intestinal cells of *glp-1(e2141)* animals treated with or without 5 mM chloroquine on adult day 1. Knockdown was conducted from egg onward. Each arrow indicates VENUS::LGG-1 puncta. Dach lines indicate single intestinal cells. (I) Quantification of VENUS::LGG-1 puncta in the intestinal cell shown in (H). Values represent mean ± SD (n = 30). *P* values (*ns* > 0.05, *****P* < 0.0001) were determined by two-way ANOVA with Tukey's test. (J) Representative fluorescent images of VENUS::LGG-1 puncta in body wall muscle of *glp-1(e2141)* mutants with muscle-specific RNAi, treated with or without 5 mM chloroquine on adult day 1. Knockdown was conducted from egg onward. Each arrow indicates VENUS::LGG-1 puncta. Scale bar, 20 μm. (K) Quantification of VENUS::LGG-1 puncta in body wall muscle shown in (J). Values represent mean ± SD (n = 30). *P* values (*ns* > 0.05, *****P* < 0.0001) were determined by two-way ANOVA with Tukey's test.

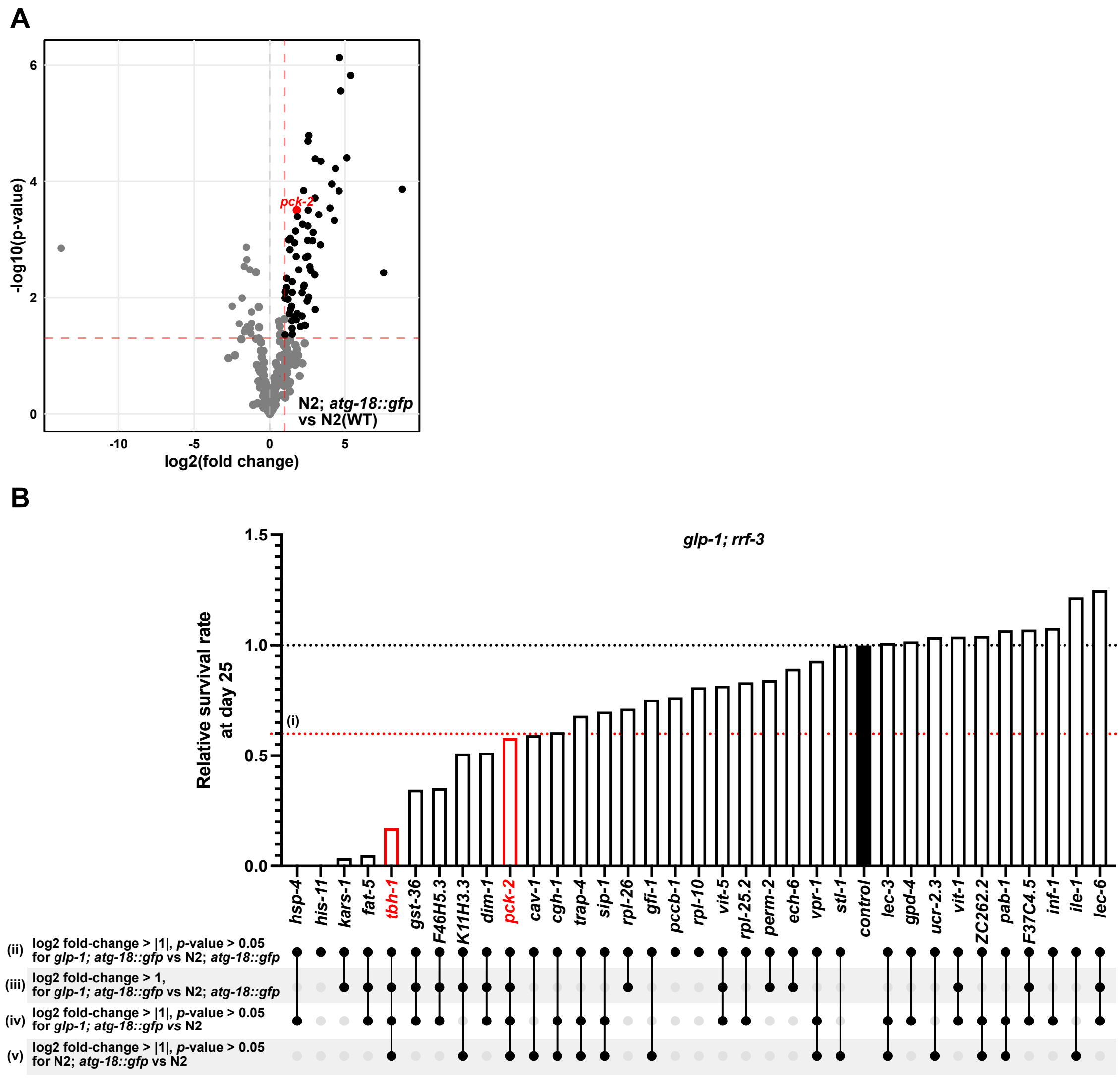

Figure S6. ATG-18 interaction profile in the context of gonadal longevity

(A) Volcano plots of ATG-18::GFP interacting proteins. Log2 fold changes are plotted against log10 p-values for N2; *atg-18::gfp* vs N2 comparisons. Proteins were considered significantly differentially expressed if $p < 0.05$ and log2 fold change > 1. The protein of interest (PCK-2) is colored red, other significant proteins are colored black, and non-significant proteins are colored gray. Red dashed line indicates the significance thresholds. (B) *glp-1; rrf-3* knockdown screens identify several candidate genes as new ATG-18 interactors required for gonadal longevity. Survival rate of 50 worms was determined at day 25 and screening conducted once. Bottom panel shows the comparison conditions under which each candidate gene was identified as an ATG-18::GFP interactor. Black circles indicate the specific comparison conditions where each gene met the criteria for candidate selection.

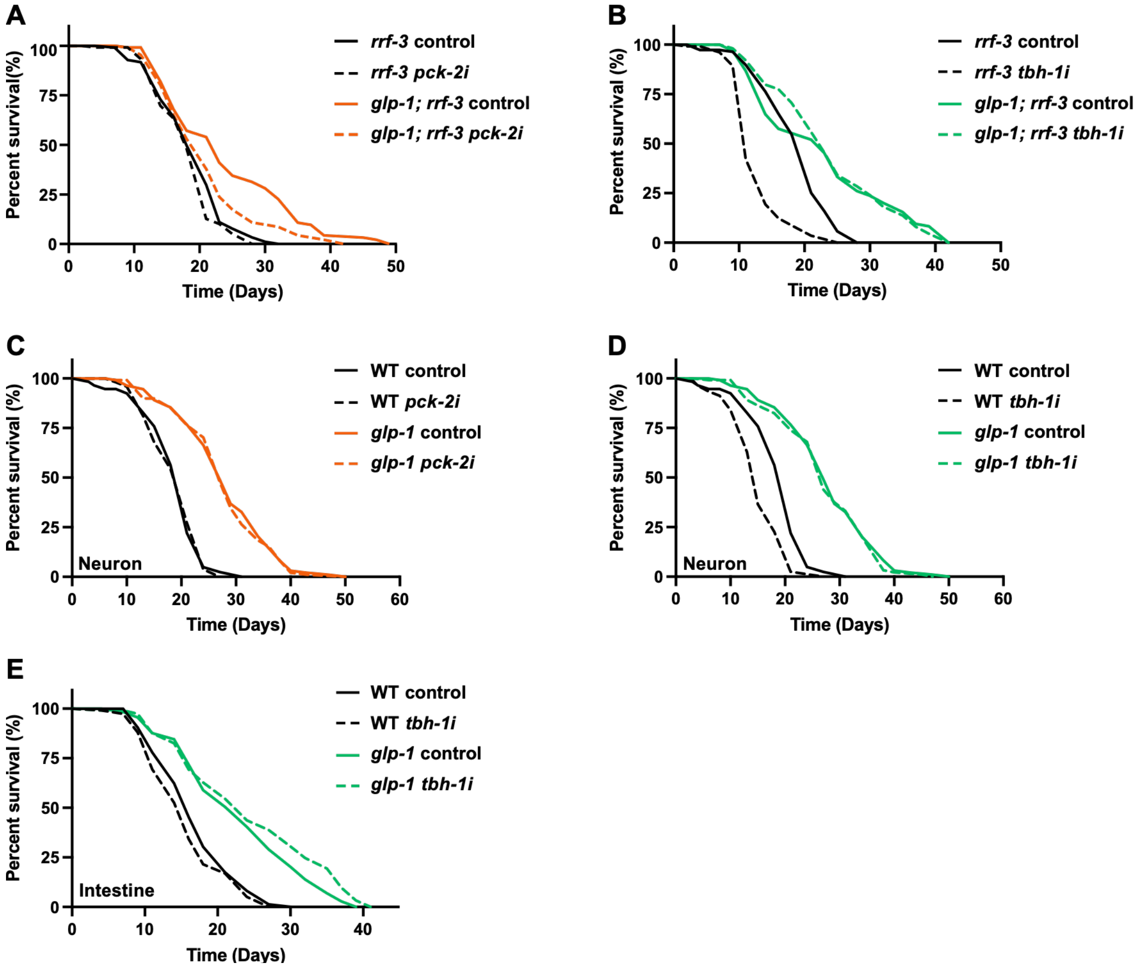

Figure S7. Knockdown of ATG-18 interacting candidates abolished extended lifespan of *glp-1* mutants.

(A and B) Lifespan analysis of wild-type (WT) and *glp-1(e2141)* animals fed bacteria expressing *luciferase* (control), (A) *pck-2*, or (B) *tbh-1* dsRNA from adult day 1. Two biological replicates were performed with 120 worms tested per condition. (C and D) Lifespan analysis of wild-type (WT) and *glp-1(e2141)* animals capable of neuron-specific RNAi (MAH677) fed bacteria expressing *luciferase* (control), (C) *pck-2* or (D) *tbh-1* dsRNA from egg onward. 120 worms were tested per condition. (E) Lifespan analysis of wild-type (WT) and *glp-1(e2141)* animals capable of intestine-specific RNAi fed bacteria expressing *luciferase* (control) or *tbh-1* dsRNA from egg onward. Two biological replicates were performed with 120 worms tested per condition.

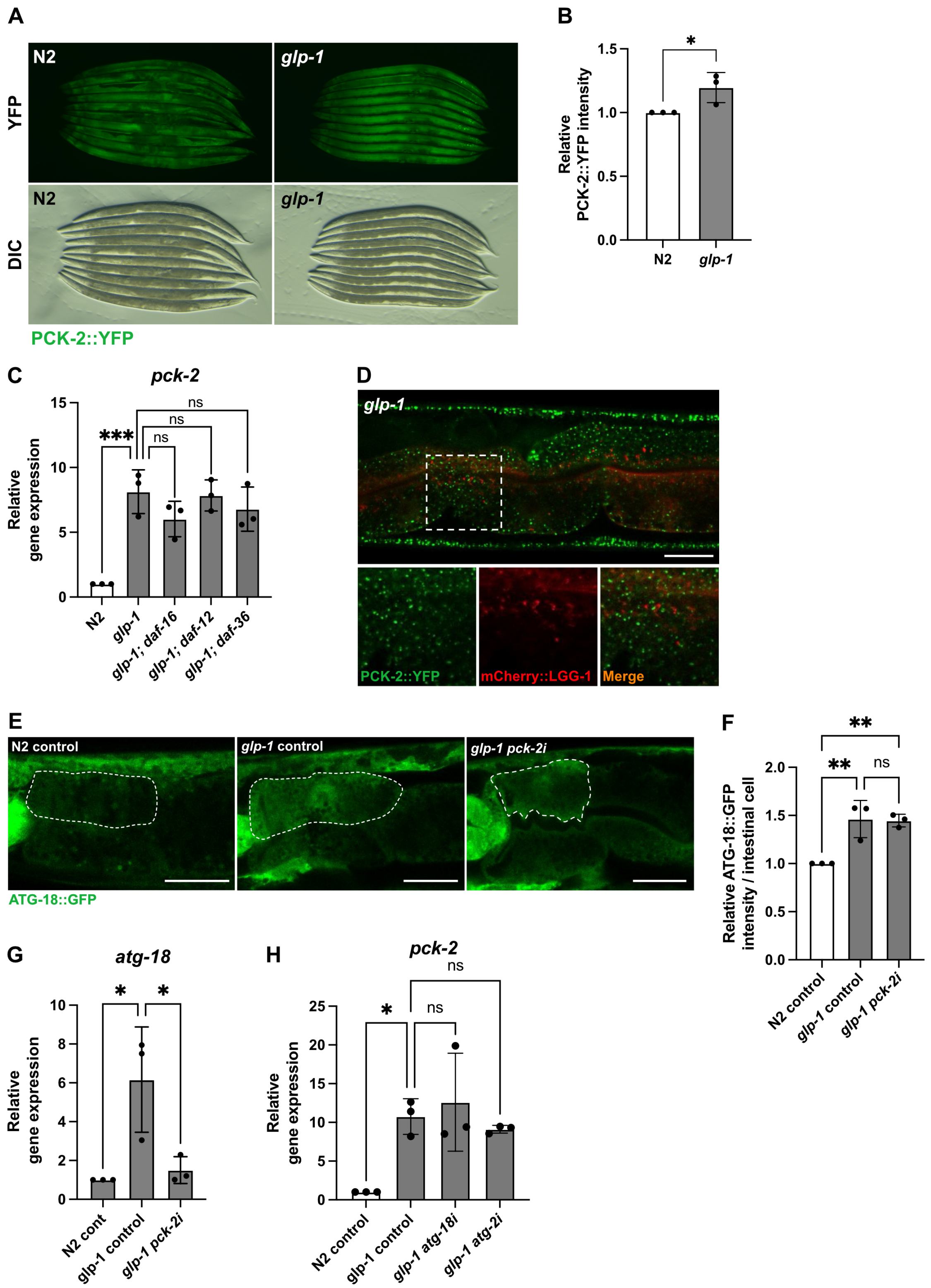

Figure S8. Germline deficiency upregulated PCK-2 expression.

(A) Representative fluorescent images of PCK-2::YFP transgenic worms in the whole body

of wild-type (WT) and *glp-1(e2141)* animals on adult day 1. Scale bars, 200 μm. (B) Quantification of PCK-2::YFP in the whole body (A). Values represent mean ± SD from three biological replicates (10 worms each). *P* value (**P* < 0.05) was determined by *t*-test. (C) qRT-PCR analysis of *pck-2* expression in wild-type (WT), *glp-1(e2141)*, *glp-1; daf-16*, *glp-1; daf-12*, and *glp-1; daf-36* animals on adult day 1. Values represent mean ± SD from three biological replicates. P values (****P* < 0.001, ns > 0.05) were determined by one-way ANOVA with Tukey's test. (D) Representative fluorescent images of PCK-2::YFP and mCherry::LGG-1 in intestinal cells of *glp-1(e2141)* animals on adult day 1, showing no colocalization between PCK-2 and LGG-1 puncta. Scale bar, 20 μm. Dashed box indicates magnified region shown below. (E) Representative fluorescent images of ATG-18::GFP in intestinal cells of wild-type (WT) and *glp-1(e2141)* animals fed bacteria expressing luciferase (control) or *pck-2* dsRNA from egg. Scale bars, 20 μm. Dashed lines indicate the intestinal cell. (F) Quantification of ATG-18::GFP in the intestinal cells shown in (F). Values represent mean ± SD from three biological replicates (10 worms each). P values (***P* < 0.01, ns > 0.05) were determined by one-way ANOVA with Tukey's test. (G) qRT-PCR analysis of *atg-18* expression in wild-type (WT) and *glp-1(e2141)* animals fed bacteria expressing luciferase (control) or *pck-2* dsRNA on adult day 1. Values represent mean ± SD from three biological replicates. P values (**P* < 0.05) were determined by one-way ANOVA with Tukey's test. (H) qRT-PCR analysis of *pck-2* expression in wild-type (WT) and *glp-1(e2141)* animals fed bacteria expressing luciferase (control), *atg-18* dsRNA, or *atg-2* dsRNA on adult day 1. Values represent mean ± SD from three biological replicates. P values (**P* < 0.05, ns > 0.05) were determined by one-way ANOVA with Tukey's test.
